# Limited Song Mixing Without Genomic Gene Flow in a Contact Zone Between Two Songbird Species

**DOI:** 10.1093/molbev/msad053

**Published:** 2023-03-03

**Authors:** Lei Wu, Jing Dang, Linfang Tang, Yalin Cheng, Gang Song, Yuehua Sun, Jochen Martens, Martin Päckert, Per Alström, Dezhi Zhang, Chenxi Jia, Fumin Lei

**Affiliations:** Key Laboratory of Zoological Systematics and Evolution, Institute of Zoology, Chinese Academy of Sciences, Beijing, China; University of Chinese Academy of Sciences, Beijing, China; Key Laboratory of Zoological Systematics and Evolution, Institute of Zoology, Chinese Academy of Sciences, Beijing, China; Key Laboratory of Zoological Systematics and Evolution, Institute of Zoology, Chinese Academy of Sciences, Beijing, China; Key Laboratory of Zoological Systematics and Evolution, Institute of Zoology, Chinese Academy of Sciences, Beijing, China; Key Laboratory of Zoological Systematics and Evolution, Institute of Zoology, Chinese Academy of Sciences, Beijing, China; Key Laboratory of Animal Ecology and Conservation Biology, Institute of Zoology, Chinese Academy of Sciences, Beijing, China; Institut für Organismische und Molekulare Evolutionsbiologie (iomE), Johannes Gutenberg Universität, Mainz, Germany; Senckenberg Naturhistorische Sammlungen, Dresden, Germany; Key Laboratory of Zoological Systematics and Evolution, Institute of Zoology, Chinese Academy of Sciences, Beijing, China; Animal Ecology, Department of Ecology and Genetics, Evolutionary Biology Centre, Uppsala University, Uppsala, Sweden; Key Laboratory of Zoological Systematics and Evolution, Institute of Zoology, Chinese Academy of Sciences, Beijing, China; Key Laboratory of Zoological Systematics and Evolution, Institute of Zoology, Chinese Academy of Sciences, Beijing, China; Key Laboratory of Zoological Systematics and Evolution, Institute of Zoology, Chinese Academy of Sciences, Beijing, China; University of Chinese Academy of Sciences, Beijing, China; Center for Excellence in Animal Evolution and Genetics, Chinese Academy of Sciences, Kunming, China

**Keywords:** song mixing, contact zone, gene flow, hybridization, leaf warblers, cryptic species

## Abstract

Song is considered to play an important role in the maintenance of prezygotic reproductive isolation between closely related songbird species. Therefore, song mixing in a contact zone between closely related species is often considered as evidence of hybridization. The Sichuan Leaf Warbler *Phylloscopus forresti* and the Gansu Leaf Warbler *Phylloscopus kansuensis*, which diverged 2 million years ago, have formed a contact zone in the south of the Gansu Province of China, where mixed songs have been observed. In this study, we investigated the potential causes and consequences of song mixing by integrating bioacoustic, morphological, mitochondrial, and genomic data with field ecological observations. We found that the two species display no apparent morphological differences, whereas their songs differ dramatically. We demonstrated that ∼11% of the males in the contact zone sang mixed songs. Two males singing mixed song were genotyped, and both were found to be *P. kansuensis*. Despite the presence of mixed singers, population genomic analyses detected no signs of recent gene flow between the two species, although two possible cases of mitochondrial introgression were identified. We conclude that the rather limited song mixing does not lead to, or result from, hybridization, and hence does not result in the breakdown of reproductive barriers between these cryptic species.

## Introduction

Acoustic communication has evolved independently in several different clades in the animal tree of life, for example, in invertebrates (Greenfield 2016) and different clades of vertebrates (Chen and Wiens 2020). Across these greatly diverse groups of taxa, multiple studies have demonstrated a strong association between vocal and genetic divergence, which has facilitated the identification of cryptic species in insects (Henry 1985, 1998; Tishechkin 2014), amphibians (Jang et al. 2011; Wang et al. 2017), mammals (Mayer and von Helversen 2001; Filippi-Codaccioni et al. 2018), birds (Irwin, Alström, et al. 2001; Alström et al. 2015, 2016; Wei et al. 2022), and even in fishes (Horvatić et al. 2021). Song of passerine birds is known to carry phylogenetic signal, and therefore bioacoustic traits are considered informative for avian taxonomy and systematics (Alström and Ranft 2003; Rheindt et al. 2004; Päckert 2018).

Bird song is important in mate attraction and territoriality (Spector 1992; Brenowitz et al. 1997; Nowicki and Searcy 2004; Catchpole and Slater 2008; Päckert 2018), and is considered to play an important role in reproductive isolation (Gill 1998; Alström and Ranft 2003; Price 2008). In allopatric speciation, songs may diverge between geographically segregated populations as a result of selection and/or drift (Price 2008). During secondary contact, songs may diverge further through the process of reinforcement of prezygotic isolation as a result of selection against unfit hybrids (Butlin 1987; Howard 1993; Liou and Price 1994; Servedio and Noor 2003). Discrimination against foreign song may increase for the same reason, without further divergence of the songs themselves (Irwin and Price 1999). Alternatively, songs may converge, and hence become more similar, in the contact zone (Helb et al. 1985; Vabishchevich and Formozov 2010; Secondi et al. 2011; Vokurkova et al. 2013), potentially leading to increased rather than decreased interbreeding between the incipient species (De Kort et al. 2002; Qvarnström et al. 2006; Sattler et al. 2007; Cros and Rheindt 2016). Several hypotheses have been formulated to explain the presence of mixed singers and song convergence (summarized in Secondi et al. 2011), for example, misimprinting (Helb et al. 1985; Olofsson and Servedio 2008), or to reduce the costs of interspecific territoriality by improved signaling (Cody 1969, 1973).

The leaf warblers (*Phylloscopus*) in the avian family Phylloscopidae form a group of small insectivorous birds widely distributed across the Old World (Bairlein et al. 2006; Price 2010; Fjeldså et al. 2020). Despite an ancient history of differentiation, with a most recent common ancestor ∼12 Ma (million years ago, Alström et al. 2018), the leaf warbler species are often remarkably similar in appearance, making them hard to distinguish in the field (Ticehurst 1938; Bairlein et al. 2006). However, there are usually marked differences in songs between species (Martens 1980; Alström and Ranft 2003; Bairlein et al. 2006) which have been of major importance for the dramatic increase in the number of recognized species, from 52 in the mid-1980s (Watson et al. 1986) to 81 at present (Gill et al. 2022; reviews in Martens 2010; Alström et al. 2013, 2018). In leaf warblers, a well-known example of a supposed effect of song divergence on reproductive isolation is the postulated ring species Greenish Warbler *Phylloscopus trochiloides*—Two-barred Warbler *Phylloscopus plumbeitarsus* complex (Irwin 2000; Irwin, Bensch, et al. 2001; Irwin et al. 2005). Their songs vary along two clines East and West of the Qinghai-Tibet Plateau, whereas songs of terminal populations in central Siberia are so different that the two sympatric taxa do not interbreed in the northern contact zone (Irwin 2000; Irwin, Bensch, et al. 2001; Irwin et al. 2005). However, subsequent extensive studies have revealed admixed songs and clinal variation of some song parameters across the Siberian contact zone (Kovylov et al. 2012), suggesting that vocalizations might not create a strict reproductive barrier between these populations, thereby disputing the ring species pattern. Later, Peterson and Anamza (2017) strongly argued against the ring species hypothesis in favor of multiple differentiated populations along a population ring around the Tibetan plateau rather than isolation of populations by distance. This was largely confirmed in a study of genome-wide data by Alcaide et al. (2014), who found that historical breaks in gene flow have existed in more than one area around the ring, and that there has been limited asymmetric introgression between the two taxa that meet in Siberia.

**Fig. 1.**
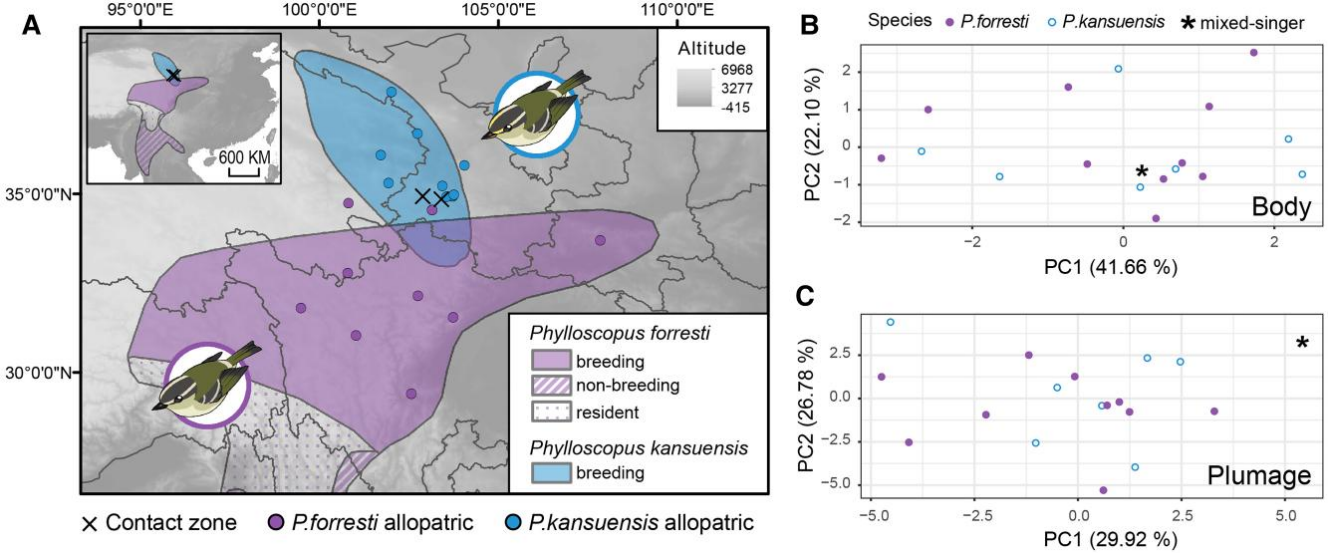
Distribution, genomic sampling sites (*n* = 35), and morphological measurements of *Phylloscopus forresti* (*n* = 10), *Phylloscopus kansuensis* (*n* = 7), and 1 mixed singer. (*A*) Distribution areas of the two species according to BirdLife International (2016a, 2016b) and genomic sampling sites in this study. Note that the overlap zone according to our study is considerably smaller and placed further north than according to BirdLife International (2016a, 2016b). The nonbreeding range of *P. kansuensis* is unknown. (*B*) Principal component analysis (PCA) based on body shape indicators. (*C*) PCA based on plumage coloration.

The Sichuan Leaf Warbler *Phylloscopus forresti* and the Gansu Leaf Warbler *Phylloscopus kansuensis* breed in mountainous regions of south central and north central China, respectively (fig. 1*A*). They were previously considered to be conspecific with each other and with Pallas's Leaf Warbler *Phylloscopus proregulus* and Lemon-rumped Warbler *Phylloscopus chloronotus*, and neither of them was even recognized at the subspecies level (Watson et al. 1986). However, Alström et al. (1997) found that they differ strongly in songs and calls, with no or very slight response to playback of songs, as well as different habitat preferences, and proposed that they should be treated as separate species. Their distinctness was later corroborated by independent analyses of songs and mitochondrial DNA (Martens et al. 2004) and by multilocus genetic analyses (Alström et al. 2018). The latter study estimated the divergence time between these two species at ∼2.4 Ma (95% highest posterior distribution ∼1.8–3.0 Ma). Alström et al. (1997) found the two species breeding only 100 km apart in southern Gansu Province, and concluded that it was likely that their ranges would overlap marginally, although they found no evidence of that.

A shared trait in the song repertoires of *P. kansuensis* and *P. forresti* is the so-called “verse song,” which is composed of short verses (strophes) of 2–5 s duration, which are separated by silent pauses (Alström and Olsson 1990; Alström et al. 1997; Martens et al. 2004). In both species, verses are composed of up to three simple trills (i.e., regular repetition of the same note) that differ in pitch, note length and repetition speed. Apart from this shared trait, *P. forresti* also has a distinct warbling song type without a clear verse structure that is given continuously for up to several minutes (Alström and Olsson 1990; Alström et al. 1997; Martens et al. 2004). This so-called “endless song” is composed of discrete units of varying length, such as trills or simple element groups or a combination of both, which are separated by pauses of irregular length. The endless song type is also given by the closely related *P. chloronotus* and *P. proregulus*, and the former of these also gives the verse song type (Alström and Olsson 1990; Alström et al. 1997; Martens et al. 2004, 2011; Martens 2010). Both endless song and verse song are given only during the breeding season, and males respond to playback of both types, showing that they are used for territorial defense (Alström and Olsson 1990; Alström et al. 1997). However, it is unknown whether both or only one of the two song types in *P. forresti* function in mate attraction.

During later, unpublished field explorations we detected locally sympatric populations of *P. forresti* and *P. kansuensis* at three sites in southern Gansu Province (Qiagai, Hezuo, and Bola; fig. 1*A*, supplementary fig. S1 and table S1, Supplementary Material online) and found some individuals displaying mixed songs. In this study, we integrate morphometrics, plumage coloration, songs, and genetic (mitochondrial and genome-wide) data to evaluate (1) the extent of song mixing in the contact zone, (2) whether mixed singers are hybrids, and (3) whether there is any evidence of ongoing gene flow between the two cryptic species.

## Materials and Methods

### Morphometrics and Plumage Color Scoring

We studied 18 specimens (10 *P. forresti*, 7 *P. kansuensis*, and 1 mixed singer; morphological data set; inferred by song types in the field) in the National Zoological Museum of China at the Institute of Zoology, Chinese Academy of Sciences, Beijing. These specimens were sampled from Gansu, Shaanxi, Qinghai, and Sichuan provinces, with 3 *P. forresti*, 4 *P. kansuensis*, and 1 mixed singer from the contact zone (Hezuo and Qiagai), respectively. We measured body weight and body length of live birds in the field. Other body shape indicators (wing length, tail length, tarsus length, and bill length) and spectrophotometric measurements were taken in the museum on the same specimens.

We used vernier calipers and electronic scales to measure body weight, body length (from tip of bill to tip of tail in a “natural pose” on its back), wing length (from wrist to tip of the longest remex in a flattened and stretched position), tail length (from tips to bases of central rectrices), tarsus length (from the hindside of the tibiotarsal joint to the joint between tarsus and middle toe), and bill length (from the tip of the bill to the leading edge of the nostril). We used ultra-low stray light fiber optic spectrometer (AvaSpec-ULS2048L-USB2; Avantes) to measure the feather reflection wavelength. Measured areas included the top of the head (the pale central stripe), back (central part), tail (upper side), throat, belly, and wings (middle part of the outermost primary remex). Each body part per specimen was measured three times with slightly different locations. Ws-2 white reference watts were used as reference signals in reflection measurement. Feather reflection was measured at a distance of 3–5 mm from the feather surface. The probe was held at 90° angle to the feather surface, and the exposure time was set to 500 ms. We set the measurement mode to Reflectance mode, and set the band interval to 1 nm. The body measurement and plumage measurement were carried out by the same person (J.D.).

According to the characteristics of the visual system of passerine birds (Hart and Hunt 2007; Ödeen et al. 2009), we retained the reflection data from 300 to 700 nm. We used “vismodel” in the package “pavo” (Maia et al. 2013) to convert the data into quantum capture values in different bands of the retina of the four-color vision system of birds, including ultraviolet wavelength *Q*_u_, long wavelength *Q*_l_, medium wavelength *Q*_m_, and short wavelength *Q*_s_. We set visual = “bluetit” and relative = FALSE. Then we used R 4.1.0 (R Core Team 2018) to run a principal component analysis (PCA) by fast.prcomp in gmodels (Warnes et al. 2018) of each body part and a combined data set with all data.

### Acoustic Sampling and Analysis

We collected sound recordings of songs of the two species from the contact zone and from allopatric areas (56 *P. forresti* and 70 *P. kansuensis* in allopatric zones, 10 *P. forresti* and 22 *P. kansuensis* in the contact zone, and 4 mixed singers; acoustic data set). Songs were recorded with a portable digital recorder (TASCAM HD-P2 and DR-100MKII) and directional microphone (RODE NTG-3). The sampling frequency of the recording was 44.1 kHz and the accuracy was set to 16 bits. To avoid repeated sampling, we only recorded one individual at each recording site (the sampling sites were at least 100 m apart).

We used Avisoft-SAS Lab Pro 5.2.08 (Avisoft Bioacoustics, Berlin, Germany) to analyze the song recordings. We set the sampling rate to 22.05 kHz, and made sonograms with frequency resolution of 43 Hz and temporal resolution of 2.90 ms (sonogram settings: Flattop window, overlap 87.5%, Fast Fourier Transform [FFT] length 512 points).

**Fig. 2.**
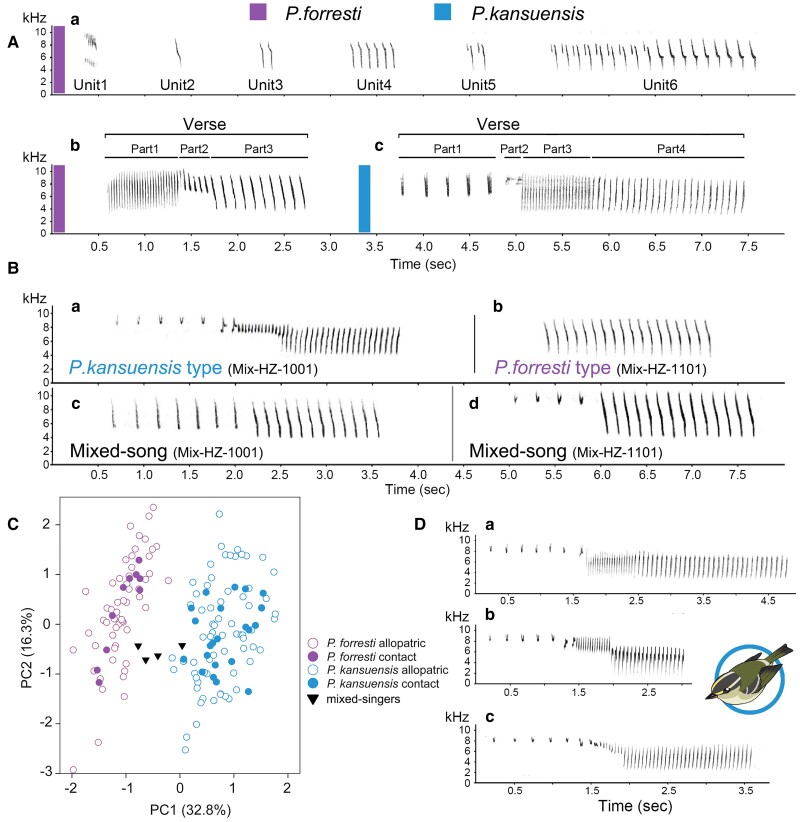
Acoustic analysis of the two leaf warbler species. (*A*) Endless song (*a*) and verse song (*b*) of *Phylloscopus forresti* and verse song of *Phylloscopus kansuensis* (*c*). (*B*) Different verses of mixed singer Mix-HZ-1001 (*a*, *c*) and Mix-HZ-1101 (*b*, *d*) in the contact zone. (*C*) PCA based on 15 log-corrected bioacoustic parameters (*n* = 162 males). (*D*) Three slightly different verse types of *P. kansuensis* from different geographical areas shown in supplementary figure S8, Supplementary Material online.

For comparison between the two species, we relied on verse songs only, because the endless song type is missing from repertoires of *P. kansuensis*. Prior to measuring, we carefully inspected sonograms of full recordings for individual variation and measured up to ten verses of each distinct verse type of a male (e.g., in repertoires of mixed singers: fig. 2*B*, Mix-HZ-1001).

For all verse songs, we took measurements from 15 bioacoustic parameters (on average 7.57 verses per individual; supplementary table S2, Supplementary Material online); frequency variables: maximum and minimum frequencies of the entire verse (*F*_max_, *F*_min_), of the first note (from here on “starting frequencies” *F*_max1_, *F*_min1_) and of the last note (from here on “terminal frequencies” *F*_max2_, *F*_min2_), frequency range of the entire verse (Δ*f*), frequency ranges of the broadest and the narrowest note type (Δ*f*_max_, Δ*f*_min_); time and structural parameters: duration of verse (*t*), of the last trill (*t*_last_) and of the longest and shortest note type (*t*_max_, *t*_min_) as well as total number of elements per verse (*n*) and in the last trill (*n*_last_). We furthermore calculated trill rate for entire verses (rate = *n*/*t*) and for the last trill of a verse (rate_last_ = *n*_last_/*t*_last_). In total, we measured 1,568 verses (*P. forresti*: verse song, *n* = 373, endless song, *n* = 349; *P. kansuensis*, verse song, *n* = 703; mixed verse song, *n* = 143). For all variables, we calculated means for (1) each individual male (*n* = 162), (2) each distinct verse type of a male (*n* = 198) and tested for significant differences between the two species and the mixed singers using a Mann–Whitney *U* test implemented in SPSS 11.5.1. For dimensionality reduction, we applied PCAs with the 15 measured song parameters using the same software with (1) individual means for each male and (2) means for each distinct verse type, separately. For analysis of intraspecific variation, we performed separate PCAs for each of the two species.

### Cytochrome *b* Sequencing and Haplotype Network Analysis

We sequenced the mitochondrial cytochrome *b* (cyt*b*) from 83 samples collected in the field (supplementary table S3, Supplementary Material online, NCBI Accession Number: OP184674 - OP184757). We extracted total DNA by tissue/cell genomic DNA extraction kit (Aidlab Biotechnologies Co., Ltd) strictly according to the manufacturer's instructions. Cyt*b* was sequenced using the primer combination L14851 (5′-AAA AAG CTT CCA TCC AAC ATC TCA GCAT GAT GAA A-3′; Kocher et al. 1989) and H15917 reverse primer (5′-TAG TTG GCC AAT GAT GAT GAA TGG GTG TTC TAC TGG TT-3′; Dietzen et al. 2003), according to polymerase chain reaction (PCR) and sequencing protocols in Päckert et al. (2014). We compiled the alignment of cyt*b* sequences using MEGA X v10.1.8 (Kumar et al. 2018) with 16 additional sequences downloaded from GenBank (supplementary table S3, Supplementary Material online). We constructed a median-joining haplotype network with PopArt v1.7 (Leigh and Bryant 2015) for 99 sequences (cyt*b*99 data set) of 758 base pair (bp) length.

### Genomic DNA Sampling and Sequencing

Muscle or blood of *P. forresti* (*n* = 19), *P. kansuensis* (*n* = 14), and mixed singers (*n* = 2) were collected for next-generation sequencing (genomic data set, *n* = 35), which included 15 individuals from the contact zone (9 *P. forresti*, 4 *P. kansuensis*, and 2 mixed singers, fig. 1*A*, supplementary table S1, Supplementary Material online; inferred by their song type a priori) and 10 individuals per species from the allopatric areas. After dehydration, the tissue samples were kept in 100% ethanol and stored at −80 °C. The geographical distributions of both species were downloaded from the IUCN (iucnredlist.org; BirdLife International 2016a, 2016b). We used ArcGIS 10.5 to visualize the distribution and sampling points. This map is only a rough illustration that does not reflect the detailed distributions, and the region where the two species overlap according to the BirdLife International data is located south of the real contact zone, and is much larger than presently known. Besides, it only shows the breeding area for *P. kansuensis* whose nonbreeding area is poorly known (the only winter records are from Guangxi in southern China and neighboring parts of northern Vietnam; Martinez and Kayser 2018).

DNA libraries with 350 bp insertions were sequenced using Illumina Nova-SEQ6000-X15 with a paired-end read length of 150 bp. We filtered the raw sequencing data based on standard procedures including (1) removing the adapters, (2) removing the low-quality reads, and (3) removing the reads where N (unknown base) accounts for >3%. The DNA library construction, sequencing, and initial sequence quality control were carried out in Berry Genomics (Beijing, China). The raw sequence data were uploaded at the NCBI Sequence Read Archive under BioProject: PRJNA865390.

We selected the genome of the Wood Warbler *Phylloscopus sibilatrix* (unpublished data, Jarvis 2016) as reference and used BWA 0.7.12 (Li and Durbin 2009) to map clean reads onto the reference genome. Samtools 0.1.19 (Li et al. 2009) was used for format conversion and operation of the sam and bam files. After filtering the alignments and removing PCR duplication, we used bcftools (Li et al. 2009) and samtools to detect single-nucleotide polymorphisms (SNPs) and used vcftools 0.1.12b (Danecek et al. 2011) and bcftools (Li et al. 2009) to filter the data. The filtering criteria include (1) quality score ≥30, (2) biallelic sites, (3) coverage ≥2, and (4) mononucleotide. Next, we used Beagle 1.4 (Browning and Browning 2007) to phase the data and calculate *r*^2^ by VCFtools 0.1.12b (Danecek et al. 2011).

### Phylogenetic and Population Genetic Analysis

We used MITObim 1.8 (Hahn et al. 2013) to assemble the mitochondrial genomes from the resequenced genomes (*n* = 35). The complete mitochondrial genome of Yellow-browed Warbler *Phylloscopus inornatus* (Anhui, China, Accession: NC_024726.1, GI: 674840194, Qing et al. 2015) was used as the reference and outgroup. To eliminate the effect of rare sequences on the assembly, we converted heterozygous sites in mitochondrial sequences into homozygous sites by miraconvert in MIRA 4.0.1 (Chevreux 2005). The mitochondrial genomes were annotated through MIOTS WebServer (mitos.bioinf.uni-leipzig.de/index.py; Bernt et al. 2013). Due to varying sequencing depth and sample type (muscle or blood), not all samples produced the complete mitochondrial genomes and not all mitochondrial genes were completely assembled (without gaps) in all mitochondrial genomes. Therefore, only the genes shared by all individuals with mitochondrial genomes were used to construct a phylogenetic tree by Bayesian Inference using BEAST 2.4.5 (Bouckaert et al. 2014). We set the priors = Birth Death Model, site model = GTR + G, MCMC chain length = 10,000,000, sampling frequency = 1,000.

To obtain a time tree, we selected cyt*b* as the marker gene from assembled mitochondrial genomes and added sequences from the three most closely related species (based on Alström et al. 2018) from GenBank (supplementary table S4, Supplementary Material online; total *n* = 48; cyt*b*48 data set). We set the parameters as follows: (1) priors = Birth Death Model, (2) site model = GTR + G, (3) substitution rate = 2.1% per site per My (Weir and Schluter 2008), (4) sampling frequency = 1,000, (5) chain length = 10,000,000. The burnin was determined by checking the parameter effective sample size and shape of the trace plot in Tracer v1.7.2 (Rambaut et al. 2018).

The population genomic studies were based on 35 individuals. The concatenated genomic SNPs were used to infer a maximum likelihood (ML) tree in iqtree2 (Minh et al. 2020) with 1,000 bootstraps. We used the parameter “–seqtype DNA -m TEST + ASC” to perform ModelFinder and subsequent tree inference. The phylogenetic trees were visualized and edited in FigTree 1.4.4 (github.com/rambaut/figtree/releases) and itol (itol.embl.de; Letunic and Bork 2021).

We used FRAPPE 1.1 (Tang et al. 2005) and Admixture 1.3.0 (Alexander et al. 2009) to estimate the population structure, with *K* values set a priori to 2–4, and 10,000 iterations. GCTA 1.24 (Yang et al. 2011) and PLINK 1.9 (Purcell et al. 2007) were used for PCA analysis. The results were visualized in R 4.1.0 (R Core Team 2018) by ggplot2 (Wickham 2016). Basic population statistics, including Fst (Wright 1931, 1951), nucleotide diversity (pi), and individual heterozygosity, were calculated by VCFtools 0.1.12b (Danecek et al. 2011). We also used the script popgenWindows.py (Merrill et al. 2019) to calculate pi, Fst, and dxy (absolute nucleotide divergence among two populations) of different genomic regions through a sliding window method. We set the sliding window size to 50 kb and the step size to 20 kb. We used the top 20 longest scaffolds for visualization in R (R Core Team 2018) by ggplot2 (Wickham 2016) and qqman (Turner 2018). To detect potential gene flow among the populations in the contact zone, we used 5 *Phylloscopus affinis* individuals (supplementary table S5, Supplementary Material online, Zhang, Tang, et al. 2019) as outgroup and calculated ABBA-BABA statistics by admixr (Petr et al. 2019). To identify the potential introgression of local regions of the genome, we removed the scaffolds smaller than 100 kb and used ABBABABAwindows.py (Martin et al. 2015) to scan the genome by 100 kb windows with the minimum count of informative sites as 100.

The linkage disequilibrium analysis was performed in PopLDdecay (Zhang, Dong, et al. 2019) with the maximum distance as 20 kb. We compressed and indexed the vcf file using bgzip and tabix, and then calculated the population demography of each species in smc++ (Terhorst et al. 2017), respectively. We set the mutation rate as 3.3e-9 (Zhang et al. 2014) and generation time as 0.948756 years per generation (average between *P. forresti* 0.929281 and *P. kansuensis* 0.968231 according to Bird et al. 2020).

## Results

### Limited Morphological Differentiation

The morphological data set included 6 body shape indicators and weight measurements obtained from 18 specimens (10 *P. forresti*, 7 *P. kansuensis*, and 1 mixed singer; supplementary table S6, Supplementary Material online). PCA results showed no obvious differentiation in body shape between *P. forresti* and *P. kansuensis* (fig. 1*B*). The first principal component (PC1) explained 41.7% of the variation, with the tail length having the highest loading value (0.58).

A total of 24 feather reflection data from each of 18 specimens were obtained in this study (supplementary table S7, Supplementary Material online). In the scatter plot constructed by PC1 and PC2, the data points of the two species greatly overlapped (fig. 1*C* and supplementary fig. S2, Supplementary Material online). Altogether, the results of body shape, weight, and plumage coloration measurements did not show any obvious morphological differentiation between *P. forresti* and *P. kansuensis*.

### Pronounced Song Differentiation

The acoustic data set included songs from 162 males in Sichuan, Qinghai, Gansu, and Shaanxi Provinces (table 1 and supplementary table S8, Supplementary Material online).

**Table 1.** Number of Individuals Included in the Song Analysis.

	Allopatric zone	Contact zone
*P. forresti*	56	10
*P. kansuensis*	70	22
Mixed singers	–	4
Total	126	36

In allopatric zones, we identified two song types of *P. forresti*, including endless song (fig. 2*Aa*) and verse song (fig. 2*Ab*). The endless song varied in the number and type of units (fig. 2*Aa* and supplementary fig. S3*g*–*m*, Supplementary Material online), and showed greater variation compared with verse songs in the PCA. In the PCA scatterplot (supplementary fig. S4, Supplementary Material online), endless songs and verse songs were separated along PC1 (that explained 82.0% and showed greatest loadings for duration (*t*) and minimum element range (Δ_fmin_); log-corrected data). Units of endless song consisted of trills (supplementary fig. S3*g*, Supplementary Material online), simple element groups (supplementary fig. S3*h*, Supplementary Material online), a combination of trills and simple elements (supplementary fig. S3*i*, Supplementary Material online), as well as a combination of two or three differently repeated trills (supplementary fig. S3*k* and *m*, Supplementary Material online). The units differed by length and were separated by pauses of irregular length. All elements descended steeply in frequency. Some of the elements had small angles upward or/and downward at the ends (supplementary fig. S3*j* and *m*, Supplementary Material online), whereas some had a gently rounded “foot” or/and angular “knee” (supplementary fig. S3*j* and *l*, Supplementary Material online). The average frequency ranged from 4.34 to 8.40 kHz and the length of units varied from 0.24 to 2.86 s.

The verse song of *P. forresti* (fig. 2*Ab* and supplementary fig. S3*a*–*e*, Supplementary Material online) consisted of clear-cut verses with three different trill parts, which included (1) trills with high trill rate (i.e., a high number of elements per unit), (2) transitional notes, and (3) trills with lower trill rate than the first part (i.e., fewer number of elements per unit). All three parts differed by length (number of elements), and all individual elements descended in frequency. In most cases, the three parts shared approximately the same maximum frequency. Individual variation was noted in the minimum frequency of the first part and in the frequency range of the second part (supplementary fig. S3*d*, Supplementary Material online) as well as in the combination of trills and notes with angles (supplementary fig. S3*a*–*c*, Supplementary Material online) or smoothly rounded “foot” (supplementary fig. S3*e*, Supplementary Material online) in the second part. The third part often contained one or more steep, descending broad-band elements, sometimes with an angle downward or rounded “foot” (supplementary fig. S3*d* and *e*, Supplementary Material online). The average minimum frequency was 4.09 kHz, and the average maximum frequency was 8.80 kHz. The length of verses ranged from 1.46 to 4.47 s with an average duration 2.68 s.

Verse song of *P. kansuensis* was markedly different from the verse song of *P. forresti* (and even more different from the endless song type of *P. forresti*). It consisted of clear-cut verses with four different parts (fig. 2*Ac* and supplementary fig. S5, Supplementary Material online), including (1) a series of high-frequency introductory elements, (2) transitional notes, (3) trills with higher mean frequency (Hz) and higher trill rate (i.e., a high number of elements per unit), and (4) trills with lower mean frequency and lower trill rate than the third part. The four parts differed by length and element types. In contrast to *P. forresti*, all elements in the third and fourth parts in verses of *P. kansuensis* were ascending in frequency. There were three element types in the first part: (1) small V-shaped elements opened upward, (2) steep-line descending elements, and (3) trills with inverted V-shaped elements. Elements in the three other parts showed great variation. The mean frequency ranged from 4.19 to 9.16 kHz and the length of verse ranged from 1.86 to 4.43 s. Verse songs from allopatric populations of *P. kansuensis* and *P. forresti* differed significantly in all parameters (Mann–Whitney *U* test, *P* < 0.001 except for *t*_last_*P* ≤ 0.01) except total number of elements. In contrast, verse songs from the contact zone differed by 13 parameters among the two species (Mann–Whitney *U* test, *P* < 0.001 except for Δ*f*_max,_*P* < 0.05; *n*, *F*_min_, Δ*f*_min_, and *t*_last_ not significant).

In the contact zone, the verse songs of *P. forresti* were like those of individuals in the allopatric zone (except for significant differences in *F*_min_ and Δ*f*_min_, Mann–Whitney *U* test, *P* < 0.05). In *P. kansuensis*, there was some variation in the verse songs that included rapidly repeated syllables of two narrowly spaced elements in the third part (supplementary fig. S5*f–h*, Supplementary Material online). We found four individuals (Mix-HZ-1001, Mix-HZ-1101, Mix-HZ-1116, and Mix-QG-8118) that displayed mixed songs in the contact zone (fig. 2*B*), which accounted to 11.1% of all recorded individuals from the contact zone (table 1). One individual (Mix-HZ-1001) displayed both complete verse song of *P. kansuensis* (fig. 2*Ba*) and a combination of the first verse part of *P. kansuensis* and endless song of *P. forresti* (fig. 2*Bc*). Another individual (Mix-HZ-1101) displayed both complete verse song of *P. kansuensis* and mixed songs with similar units and verses as in Mix-HZ-1001 (fig 2*Bd*), as well as units of endless song of *P. forresti* (fig. 2*Bb*). The other individuals had similar song patterns with complete verses of *P. kansuensis* and a combination of *P. kansuensis* and *P. forresti* (supplementary fig. S6, Supplementary Material online). If we treated typical *P. kansuensis* verse songs as conspecific songs, mixed songs, and *P. forresti* songs as heterospecific songs, the proportion of heterospecific songs reached a stable proportion after the first 10 recordings counted, accounting for about 40–60% of the total songs (supplementary table S8 and fig. S7, Supplementary Material online). The average maximum frequency was 8.99 kHz and the average minimum frequency 4.53 kHz. Duration of verses ranged from 2.44 to 2.53 s.

Verse songs of *P. forresti* and *P. kansuensis* were well differentiated in PCA and appeared as separate clusters in the scatterplot of PC1 versus PC2 (fig. 2*C*). PC1 explained 32.8% of the total variation, and starting frequencies (*F*_max_, *F*_max1_, *F*_maxlast_) and frequency range (Δ*f*, *F*_min1_) rate showed the highest factor loadings, that is, the pitch of songs increased with increasing values on PC1 (supplementary table S9, Supplementary Material online). The speed of the last trill differed significantly between the two species, with *P. kansuensis* displaying more rapid trills than *P. forresti* (supplementary table S8, Supplementary Material online). Mixed singers displayed verse songs with intermediate speed of the last trill, and their songs differed significantly from all other populations (within and beyond the contact zone; fig. 2*C* and supplementary table S8, Supplementary Material online). PC2 explained another 16.3% of the total variation (a cumulative 49.1%), and duration of longest element (Δ*f*_max_) and other time parameters like the time of the last trill (*t*_last_) had the highest factor loadings, that is, length of song units increased with increasing values on PC2. Admixed songs differed from *P. forresti* songs from the contact zone and areas beyond by 10 and 12 parameters, respectively, and from *P. kansuensis* songs from the contact zone and areas beyond by 6 and 7 parameters, respectively (supplementary table S8, Supplementary Material online).

We furthermore identified three slightly different verse types of *P. kansuensis*: type A in the western part of the distribution (fig. 2*Da*), type B in the southern contact zone (fig. 2*Db*), and type C in the eastern part (fig. 2*Dc*). These verse types differed in the last two parts of a verse (parts 3 and 4). The distributions of types B and C were relatively narrow at the edge of the distribution of *P. kansuensis* in Gansu province (supplementary fig. S8, Supplementary Material online). Songs of *P. kansuensis* from the contact zone differed significantly from those from allopatric populations in 7 out of 15 parameters (Mann–Whitney *U* test, *P* < 0.01 for *t*, Δ*f*_min_, *F*_max1_; *P* < 0.05 for *t*_min_, *n*, *n*_last_, trill rate). These differences do not define dialects, because one male could have both types (e.g., male Kan-QL-1201). Also, mixed singers sang both variants (supplementary fig. S9*B* and *C*, Supplementary Material online).

### Concordance Between Song Type and Genotype

**Fig. 3.**
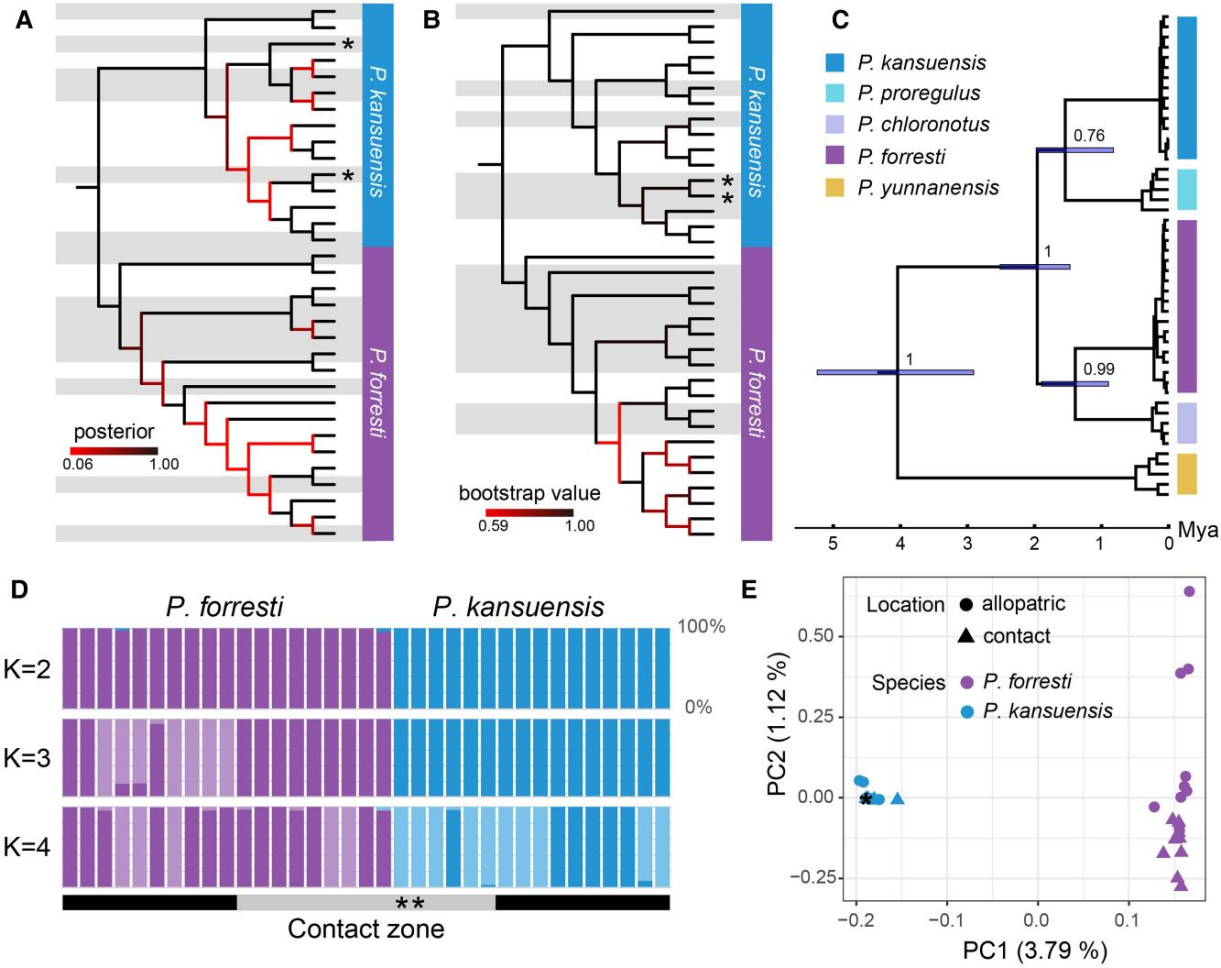
Phylogenetic trees and population structure of *Phylloscopus forresti* and *Phylloscopus kansuensis*. (*A*) Cladogram inferred by Bayesian Inference from 11 mitochondrial genes (cyt*b*, *atp6*, *atp8*, *cox1*, *cox2*, *cox3*, *nad1*, *nad2*, *nad3*, *nad4*, *nad4l*) in BEAST2. Note that two of the samples failed to assemble the mitochondrial genomes, thus 33 individuals were included in this analysis (vs. 35 in the analyses based on SNP data). (*B*) Cladogram inferred by ML from 2,484,080 informative genomic SNPs of 35 individuals in iqtree2 with best-fit model K3Pu + F + ASC + G4. (*C*) Mitochondrial tree inferred from partial cyt*b* sequences (*n* = 48, cyt*b*48 data set; target species: supplementary table S1, Supplementary Material online; close relatives: supplementary table S4, Supplementary Material online) with BEAST2. Posterior probabilities are labeled at the nodes. (*D*) Results of population structure analysis by FRAPPE based on 11,383,944 genomic SNPs inferred from the genomic data set (*n* = 35). The samples from the allopatric regions are located on both sides of the figure, while the samples from the contact zone are located in the middle. (*E*) Scatter plot from PCA of 11,383,944 genomic SNPs from the genomic data set (*n* = 35). Individuals from the contact zone are indicated by gray background in *A*, *B*, and *D*, and mixed singers are marked with asterisks in *A*, *B*, *D*, and *E*.

We resequenced the genome of 35 individuals covering the contact zone (9 *P. forresti*, 4 *P. kansuensis*, and 2 mixed singers; inferred by their song type a priori) and allopatric areas (10 *P. forresti* and 10 *P. kansuensis*), producing a total of 360 gigabase (GB) of sequence data with an average of 10.3 GB per individual. The mean coverage was 95.2% and the mean sequencing depth 11.2× (supplementary table S1, Supplementary Material online).The tree based on 11 mitochondrial genes for 33 samples (FOR-QG-8125 and KAN-LT-1555 failed to assemble the mitochondrial genomes) recovered both species as monophyletic (fig. 3*A*). Two mixed singers (Mix-HZ-1116 and Mix-QG-8118) from the contact zone had *P. kansuensis* mitochondrial DNA (fig. 3*A*). The same results were obtained from the tree reconstructed by genome-wide SNPs for 35 samples (fig. 3*B*). Both the nuclear and mitochondrial cladograms show that individuals from the contact zone are dispersed across the tree (fig. 3*A* and *B*), indicating a lack of differentiation between the contact zone and allopatric populations.

According to the cyt*b* tree based on 48 sequences (cyt*b*48 data set: 33 individuals in supplementary table S1, Supplementary Material online and 15 additional close relatives in supplementary table S4, Supplementary Material online), *P. forresti* and *P. kansuensis* diverged about 2 Ma (1.96 Ma, 95% HPD = 1.47–2.51 Ma; fig. 3*C*). The two species are not sister species, supporting the results of Martens et al. (2004), Päckert et al. (2012), and Alström et al. (2018), which were based on mitochondrial sequences in the two earlier studies, and on mitochondrial and nuclear loci in the latter study.

Strong divergence of mitochondrial lineages was also reflected by the cyt*b* haplotype network based on 99 sequences (cyt*b*99 data set: 83 samples in this study and 16 additional sequences from GenBank; supplementary fig. S10, Supplementary Material online). The star-like *P. kansuensis* cluster contained eight haplotypes (the central one shared by 30 individuals) and was separated from *P. forresti* by a minimum of 22 substitutions (and from *P. chloronotus* and *P. proregulus* by 24 and 19 substitutions, respectively). The *P. forresti* cluster was more structured and more diverse with 18 haplotypes (the most frequent 1 found in 23 individuals) and was separated from *P. proregulus* and *P. chloronotus* by a minimum of 21 and 32 substitutions, respectively. We found a strong match between phenotype and cyt*b* haplotype, except for two birds that sang *P. kansuensis* song (1,130 from Hezuo in the sympatric zone, and 1,105 from Lianhuashan, which is in an area where only *P. kansuensis* is known to occur), which carried a *P. forresti* haplotype, suggesting gene flow and introgression of mitochondria.

The results of population structure analysis based on 11,383,944 SNPs inferred from the genomic data set (*n* = 35) showed that the most strongly supported *K*-value was 2 (CV error = 0.40498, the minimum value among all results). The samples were divided into two clusters according to species, without any further distinction among individuals from the contact zone or allopatric areas (fig. 3*D*). Notably, there was hardly any evidence of admixture between the two species as the *K*-value increased (fig. 3*D*). The PCA also showed significant differentiation between *P. forresti* and *P. kansuensis* on PC1, which explained 3.79% of the total variation (fig. 3*E*). For *P. kansuensis*, individuals from the contact zone showed great overlap with noncontact zone individuals without significant genetic differentiation, whereas *P. forresti* individuals from the contact zone were partly separated from those in allopatric areas along PC2, which explained another 1.12% of the total variation (fig. 3*E*). Both the mitochondrial and genomic data supported concordance between song type and genotype. The two mixed singers were pure *P. kansuensis* according to the population structure analyses (fig. 3).

### Lack of Recent Gene Flow

Based on the above results, we treated the two genotyped mixed singers as *P. kansuensis* in the following analysis and classified them into the *P. kansuensis* contact zone population (*n* = 6, with 4 *P. kansuensis* and 2 mixed singers). The nucleotide diversity represented by average pi of 100 kb windows of *P. forresti* (0.000378) was slightly higher than that of *P. kansuensis* (0.000365, table 2), consistent with the more dispersed distribution of the former along PC2 (fig. 3*E*). The average individual heterozygosity value was slightly higher in *P. forresti* (−0.080402) compared with that of *P. kansuensis* (−0.080924, table 2).

**Table 2.** Genomic Diversity of the Populations in Contact Zone and Allopatric Areas Based on the Genomic Data Set (*n* = 35).

	*P. f*	*P. k*	*P. f* cont	*P. f* allo	*P. k* cont	*P. k* allo
**pi**	0.000378	0.000365	0.000382	0.000379	0.000373	0.000366
***F***	−0.080402	−0.080924	−0.081459	−0.076067	−0.076643	−0.079566

Note.—pi is nucleotide diversity, and F is the heterozygosity. *P. f* stands for *P. forresti* and *P. k* for *P. kansuensis*, respectively, and the suffix is a contraction of contact zone (cont.) or allopatric areas (allo).

We next compared Fst between the sympatric and allopatric populations as a measure of the degree of population differentiation. The average Fst between *P. kansuensis* and *P. forresti* was 0.0854, and the average Fst between these species in sympatry and allopatry was 0.1035 and 0.0996, respectively. The higher Fst in the contact zone indicates that the two species hardly, if at all, hybridize during secondary contact. Moreover, interspecific dxy in the contact zone (0.00147) was similar to that of the allopatric populations (0.00148). The results of the sliding window analysis (window size = 50 kb) did not discover any windows with significant differentiation between sympatric and allopatric populations (supplementary fig. S11, Supplementary Material online), and supported that *P. kansuensis* and *P. forresti* are genetically well separated throughout their ranges.

**Fig. 4.**
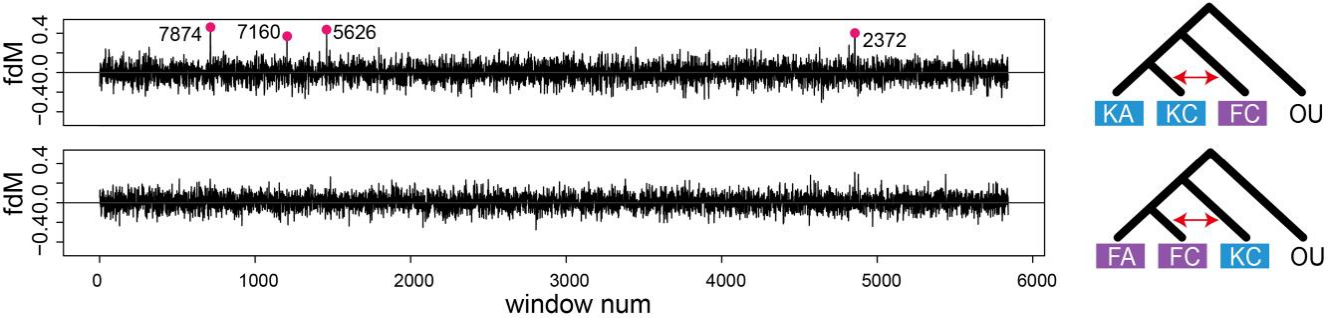
Potential ancient asymmetric gene flow between *Phylloscopus forresti* and *Phylloscopus kansuensis* inferred by fdM values of 100 kb windows in ABBA-BABA tests based on the genomic data set (*n* = 35). The models are shown by the cladograms on the right (F stands for *P. forresti*, K for *P. kansuensis*, C for contact region, A for allopatric region, and OU for outgroup) with red arrows showing the gene flow under test. Windows with high fdM are highlighted with red dots and corresponding scaffolds are labeled nearby.

Next, we used the ABBA-BABA method in admixr (Petr et al. 2019) to test for evidence of ancient gene flow (table 3). We set up two models, including test 1 (P1 = KA, P2 = KC, P3 = FC) and test 2 (P1 = FA, P2 = FC, P3 = KC), where K stands for *kansuensis*, F for *forresti*, A for allopatric areas, and C for contact zone (fig. 4). The *D* statistics were weakly positive (0.0007 and 0.0013) between the populations in the contact zone with nonsignificant *Z* scores (0.97 and 2.2) in both tests, indicating little evidence of gene flow (Korneliussen et al. 2014).

**Table 3.** ABBA-BABA Tests Among Different Populations Based on the Genomic Data Set (*n* = 35).

W	X	Y	Z	*D*	stderr	Zscore	BABA	ABBA	nsnps
K_cont	K_allo	F_cont	Outgroup	0.0007	0.0007	0.97	10,747	10,732	131,576
F_cont	F_allo	K_cont	Outgroup	0.0013	0.000588	2.2	11,052	11,023	131,595

Note.—F stands for *P. forresti* and K Stands for *P. kansuensis*. The suffix is a contraction of contact zone (cont.) or allopatric (allo.) areas.

We further scanned the genome using 100 kb windows to test the potential partial introgression between the two species (fig. 4). We found some outliers with high fdM values in test 1 ranging from 0.3735 (scaffold2372:100,001–200,000) to 0.4744 (scaffold7874:100,001–200,000), but not in test 2.

The linkage disequilibrium patterns were different between *P. forresti* and *P. kansuensis* (supplementary fig. S12, Supplementary Material online). The *R*^2^ for *P. kansuensis* in the contact zone was close to 0.15, whereas *R*^2^ values for other populations were close to 0.10 (supplementary fig. S12, Supplementary Material online). At the interspecific level, *R*^2^ of *P. kansuensis* was ∼0.02 higher than that of *P. forresti*, which implies that the current effective population size of *P. kansuensis* is probably smaller that of *P. forresti*. The population demographic reconstruction supports this hypothesis, where *P. forresti* has experienced a more dramatic expansion than *P. kansuensis* (supplementary fig. S13, Supplementary Material online), further in agreement with the much smaller breeding range of *P. kansuensis* than of *P. forresti* (fig. 1*A*).

## Discussion

Our study confirms that *Phylloscopus forresti* and *P. kansuensis* are cryptic species, which are undifferentiated in morphology (supporting Alström et al. 1997) while they have diverged markedly in song (supporting Alström et al. 1997; Martens et al. 2004; Martens 2010). We provide the first evidence of a secondary contact zone between *P. forresti* and *P. kansuensis*, which is located in southern Gansu Province in China. In the previous study, Alström et al. (1997) found the two species only 100 km apart, and hypothesized a marginal range overlap, which was later positioned in a narrow area in the Min Shan range according to projections by BirdLife International (2016a, 2016b; fig. 1*A*). However, these projections are approximations (Ramesh et al. 2017), and the confirmed sites of sympatry based on our field observations were all located north of BirdLife's predicted area of sympatry (fig. 1*A* and supplementary fig. S1, Supplementary Material online). According to our field explorations, the contact area is about 10 km wide longitudinally and 50 km wide latitudinally (supplementary fig. S1, Supplementary Material online). In that region, the forest belt is fragmented, with forest patches varying from 0.05 to 3.3 square kilometers. In this contact zone, ∼11% of the males sang a mix of the two species’ species-specific songs. Two of these mixed singers were genotyped, and found to be pure *P. kansuensis*. Based on the genomic data, we found no evidence of gene flow between the two species despite the song mixing, indicating that song mixing does not necessarily lead to, or result from, hybridization. However, we cannot exclude gene flow of mtDNA between the two species, as two birds that sang typical *P. kansuensis* song carried a *P. forresti* mtDNA haplotype, indicating potential past mtDNA introgression from *P. forresti* to *P. kansuensis*. Moreover, we detected some windows with high fdM (fig. 4), although these outliers may also be the result of incomplete lineage sorting in this region. Wider sampling and higher sequencing coverage may help to reach a more robust conclusion.

### Limited Song Mixing in the Contact Zone

There were clear-cut differences in the verse songs between the two sympatric leaf warbler populations. However, 11.1% of the males (4 out of 36) sang mixed songs, either an alternation between both species’ typical song parts or chimeric verses built up of units from both species, or both.

Song mixing between closely related species in contact zones has been widely reported (Helb et al. 1985; Vabishchevich and Formozov 2010; Secondi et al. 2011; Vokurkova et al. 2013; Shipilina et al. 2017). It has been suggested that song copying may facilitate the co-existence between closely related species by increasing the efficiency of territorial signaling directed toward heterospecific males, and to be adaptive for the dominant species in a contact zone and maladaptive for the subordinate species (“convergent agonistic character displacement”; Cody 1969; Cody 1973; Emlen et al. 1975; Robinson and Terborgh 1995; Grether et al. 2009; Tobias and Seddon 2009; Reif et al. 2015; Grether et al. 2017; Souriau et al. 2018), although this explanation has been criticized (e.g., Brown 1977; Helb et al. 1985). It is unknown whether either of the two leaf warblers in our study is dominant over the other one. Alström et al. (1997) reported significantly longer wings in *P. kansuensis* than in *P. forresti*, which might indicate that the former is dominant over the latter (Grasso et al. 1996). However, the samples of *P. forresti* measured by Alström et al. (1997) came from a geographically more extensive area than the ones in the present study, which did not differ significantly.

Among our genotyped individuals from the contact zone, two mixed singers were assigned to *P. kansuensis* based on the genome-wide SNPs, which despite the limitations of our sampling might be indicative of unidirectional song copying. Moreover, all mixed singers in our acoustic data set sang both complete *P. kansuensis* verse songs and mixed songs, but no complete *P. forresti* verse songs or endless songs. This might indicate that these birds were not *P. forresti*, although genetic data would be needed to confirm this. There are multiple examples where usually only one of the two species in an avian secondary contact zone is a mixed singer (*Luscinia* nightingales: Vokurkova et al. 2013; Reif et al. 2015; Souriau et al. 2018; *Ficedula* flycatchers: Alatalo et al. 1990; Haavie et al. 2004; Qvarnström et al. 2006; Vabishchevich and Formozov 2010; *Parus* tits: Nazarenko et al. 1999; Päckert et al. 2005; Kvist and Rytkönen 2006). In some other examples of unidirectional heterospecific song copying, the species performing mixed song varies depending on the relative abundance of the two species in different regions (*Certhia* treecreepers: Thielcke 1960, 1986; Clausen and Toft 1988; *Regulus* crests: Becker 1977). In these species, the rarer one copies the more common one (also in the Swedish *Ficedula* hybrid zone). If our indications of asymmetric song copying are correct, this is the opposite to what we found in the contact zone between *P. kansuensis* and *P. forresti*, where *P. kansuensis* was approximately two to three times more numerous than *P. forresti* (see below).

Our field song collections covered 5 years and the songs from the contact zone covered 4 years (2010–2013). In total, we recorded songs from 22 *P. kansuensis* and 10 *P. forresti*. During our expedition in 2011, which primarily focused on the contact zone, we captured 20 *P. kansuensis* in the field, but only 7 *P. forresti*. Since there was no species preference in our recording or capture efforts, the difference in the number of samples likely reflects actual demographic differences between the two species in the contact zone. These facts suggest that song mixing is not common in the contact zone. Whether song mixing is asymmetrical, as indicated by our results, needs to be verified by larger samples of genotyped mixed singers.

### Song Mixing Without Recent Genomic Gene Flow

Vocalizations are believed to act as important signals involved in mate choice in many organisms, and hence are potentially important for reproductive isolation between species (e.g., Filippi-Codaccioni et al. 2018; Campbell et al. 2019; Drillon et al. 2019; Vedenina and von Helversen 2003; Hagberg et al. 2022). In a study of *Ficedula* flycatchers, the preference for the local song dialect over other conspecific dialects and heterospecific song was found to develop already in the nestlings (Wheatcroft et al. 2022). It has been suggested that cultural evolution, in conjunction with associated learning predispositions, may drive the emergence of premating reproductive barriers, particularly in passerine birds (Mason et al. 2017; Arato and Fitch 2021; Wheatcroft et al. 2022).

Since female *P. forresti* might be attracted by the mixed songs sung by some *P. kansuensis* males, one could expect at least asymmetric gene flow in the contact area. Due to the limitations of our field work, only two mixed singers were included in the genomic data set. However, widespread hybridization and backcrossing are supposed to cause genomic mixing between two species. Accordingly, if there had been ongoing hybridization and introgression in the contact zone, we should expect to find evidence of this regardless of whether we sampled the mixed singers or not. However, we found no evidence of hybridization or introgression in the contact zone, although larger samples might have exposed such rare cases. On the other hand, the single male (KAN-HZ-1130) with *P. kansuensis* song and a *P. forresti* mitochondrial cyt*b* haplotype in the contact zone (results from cyt*b*99 data set, no genomic data available for this individual) might represent a case of interbreeding between a male *P. kansuensis* and a female *P. forresti*. However, it is possible that female hybrid sterility, in accordance with “Haldane's rule” (Haldane 1922), would reduce further introgression even if interbreeding sometimes takes place. The finding of a male with *P. forresti* cyt*b* haplotype but with the song of *P. kansuensis* (KAN-LHS-1105 from Lianhuashan; no genomic data available for these individuals) at a locality where only *P. kansuensis* is known to occur might be the result of ancient introgression, or of an out-of-range *P. forresti* copying the song of *P. kansuensis*. Larger sample size of genomic DNA is warranted to elucidate the prevalence of gene flow.

Song mixing often accompanies gene flow in contact zones of closely related birds. Four studies of *Phylloscopus* warblers, where song mixing occurs in contact zones, all indicate various levels of interspecific gene flow (Bensch et al. 2002; Alcaide et al. 2014; Marova et al. 2017; Shipilina et al. 2017). Likewise, hybridization has been found to be common in contact zones of mixed-singing *Parus* tits (Päckert et al. 2005; Kvist and Rytkönen 2006), *Ficedula* flycatchers (Haavie et al. 2004; Qvarnström et al. 2006; Vabishchevich and Formozov 2010), and *Hippolais* warblers (Secondi et al. 2003, 2011). In the recently established (probably some 70–150 years; Lundberg and Alatalo 1992) *Ficedula* contact zone in Sweden, 2–7% of the population are hybrids (Saetre and Saether 2010), whereas in an older contact zone in western Russia, hybridization is rare (<1%; Vabishchevich and Formozov 2010). In Sweden, mixed singing has been shown to promote hybridization (Qvarnström et al. 2006), while that does not seem to be the case in western Russia, where up to ∼40% of all males of one species included syllables similar to the other species in their repertoire (Vabishchevich and Formozov 2010). Although the ages of contact zones between closely related bird species are generally poorly known or completely unknown, as is the case also with the two leaf warblers of the present study, the situation in the two differently aged *Ficedula* flycatcher contact zones suggests that mixed singing may promote interbreeding at the early stages of contact, whereas it will cease with time. In such cases, where there is no evidence of increased differentiation in the vocal signals themselves, reinforcement of increased discrimination may be the main reason for the cessation of hybridization (cf. Irwin and Price 1999).

In our study, despite the presence of a few mixed singers, which displayed admixed repertoires of different verse types, the distinctness of song types (endless song and verse song, respectively) and of verse songs (*P. kansuensis* and *P. forresti* type, respectively) are largely maintained in the contact zone. This is unlike the situation in a New World warbler hybrid zone (Love and Goller 2021), where the bioacoustic traits converged.

In our study, *P. forresti* and *P. kansuensis* displayed no obvious differentiation in plumage coloration or body structure (fig. 1*B* and *C*), in agreement with Alström et al. (1997), who concluded that they were “essentially identical on plumage,” although differing on average in bare part colors, wing length, and wing formula. No behavioral differences are known, and Alström et al. (1997) reported the same threat posture and behavior in both species in response to playback of conspecific song. Accordingly, the pronounced differences in song can be hypothesized to act as a premating reproductive barrier between these two species. This may be supported by playback tests carried out by Alström et al. (1997), which found that the majority of all tested birds did not respond aggressively to playback of the other species’ song. However, field experiments by Alström et al. (1997) were limited to male birds and therefore allow for conclusions on an effect of song divergence on intra-species recognition among males. Because discrimination against heterospecific songs might differ among sexes of passerine species (Searcy and Brenowitz 1988), we cannot conclude on a potential effect of song divergence on mate choice (and thus on potential premating barriers) in our study species. Female hybrid sterility, in accordance with “Haldane's rule” (Haldane 1922), is an alternative explanation for the lack of gene flow, which needs to be tested.

Another limitation of the playback study by Alström et al. (1997) is that it was only carried out on birds in allopatry (since no sympatry was found at that time). In some other species, in which song mixing and hybridization occurs in contact zones, it has been found that birds only respond to playback of heterospecific song where they occur in sympatry but neglect heterospecific songs in allopatry (e.g., Emlen et al. 1975; Kovylov et al. 2012; Marova et al. 2017). In other words, experience with potential heterospecific competitors may be needed to trigger an agonistic response, so it cannot be eliminated that *P. kansuensis* and *P. forresti* might respond to each other's songs in sympatry and asymmetrically, as in *Henicorhina* wood wrens (Burbridge et al. 2015).

Differences in breeding habitat preferences might contribute toward reproductive isolation. Alström et al. (1997) found *P. kansuensis* to prefer deciduous forests with a low percentage of conifers, whereas *P. forresti* favored coniferous forests at higher elevation. This might explain why *P. forresti* was always present in lower numbers in the contact zone, where the habitat might be suboptimal for that species, as the forests were a mix of broadleaf and coniferous. Such subtle ecological segregation has also been confirmed for two *Luscinia* nightingale species, however, only for allotopic sites in the contact zone (i.e., where only one of the two species was present) but not for syntopic sites (Sottas et al. 2018).

## Conclusion

The two leaf warbler species *P. forresti* and *P. kansuensis* were studied with respect to morphometrics, plumage coloration, song, mitochondrial and genomic DNA, with focus on a recently found contact zone between them. No plumage or structural differences were detected. In contrast, the differences in song were pronounced. Approximately 11% of the birds in the contact zone were mixed singers which, based on studies of some other passerine birds, could have been hybrids. However, we found no evidence of introgression of nuclear DNA between the two species, although we detected two cases of possible mitochondrial introgression, one inside and one away from the contact zone. Although we cannot exclude the presence of postzygotic isolation in this system, we hypothesize that the differences in song between the two species act as a premating reproductive barrier between them, and that the rather limited song mixing does not lead to, or result from, hybridization, and that it does not result in the breakdown of this reproductive barrier.

## Supplementary Material

msad053_Supplementary_Data
