# Supplementary material for: Limited Song Mixing Without Genomic Gene Flow in a Contact Zone Between Two Songbird Species
Source: Mol Biol Evol. 2023 Mar 3;40(3):msad053. doi: 10.1093/molbev/msad053 (PMC10050365; doi:10.1093/molbev/msad053)

**Supplementary figures**

**Supplementary figure 1.** The satellite map of contact zone, including Hezuo (102.89487 E, 34.92907 N), Bola (102.85274 E, 34.83076 N), and Qiagai (103.40826 E, 34.84824 N). The distances between these three areas are shown by red lines.


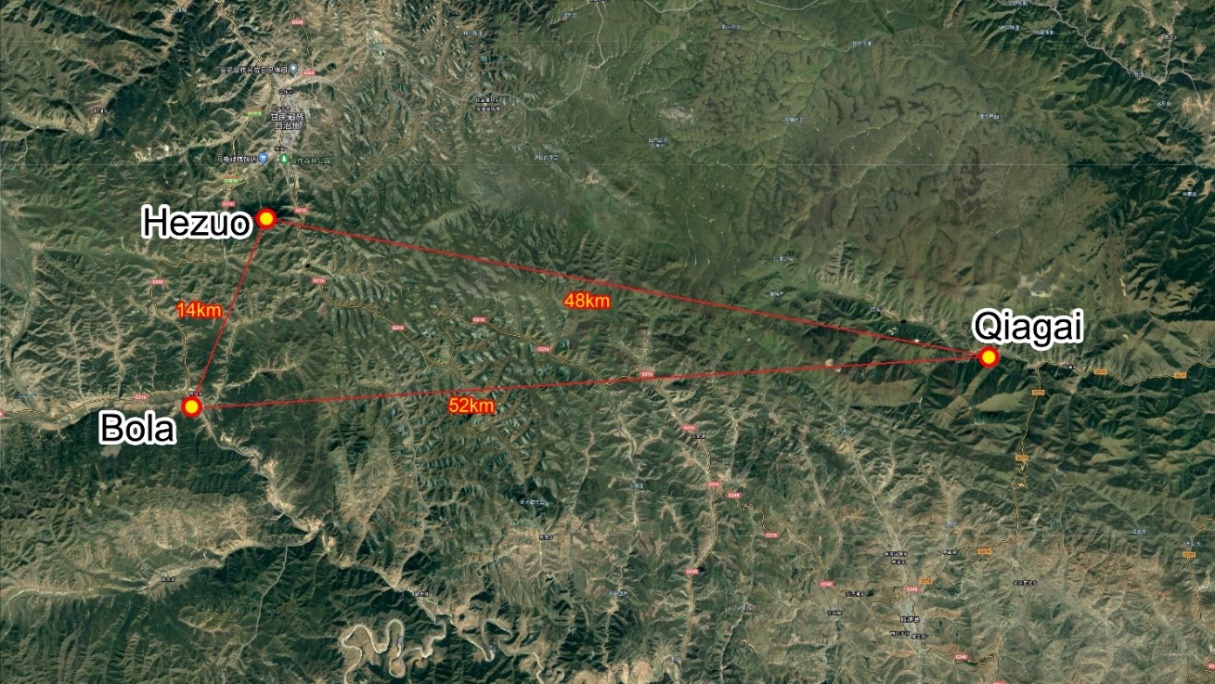


**Supplementary figure 2.** Principle component analysis based on plumage coloration.

**
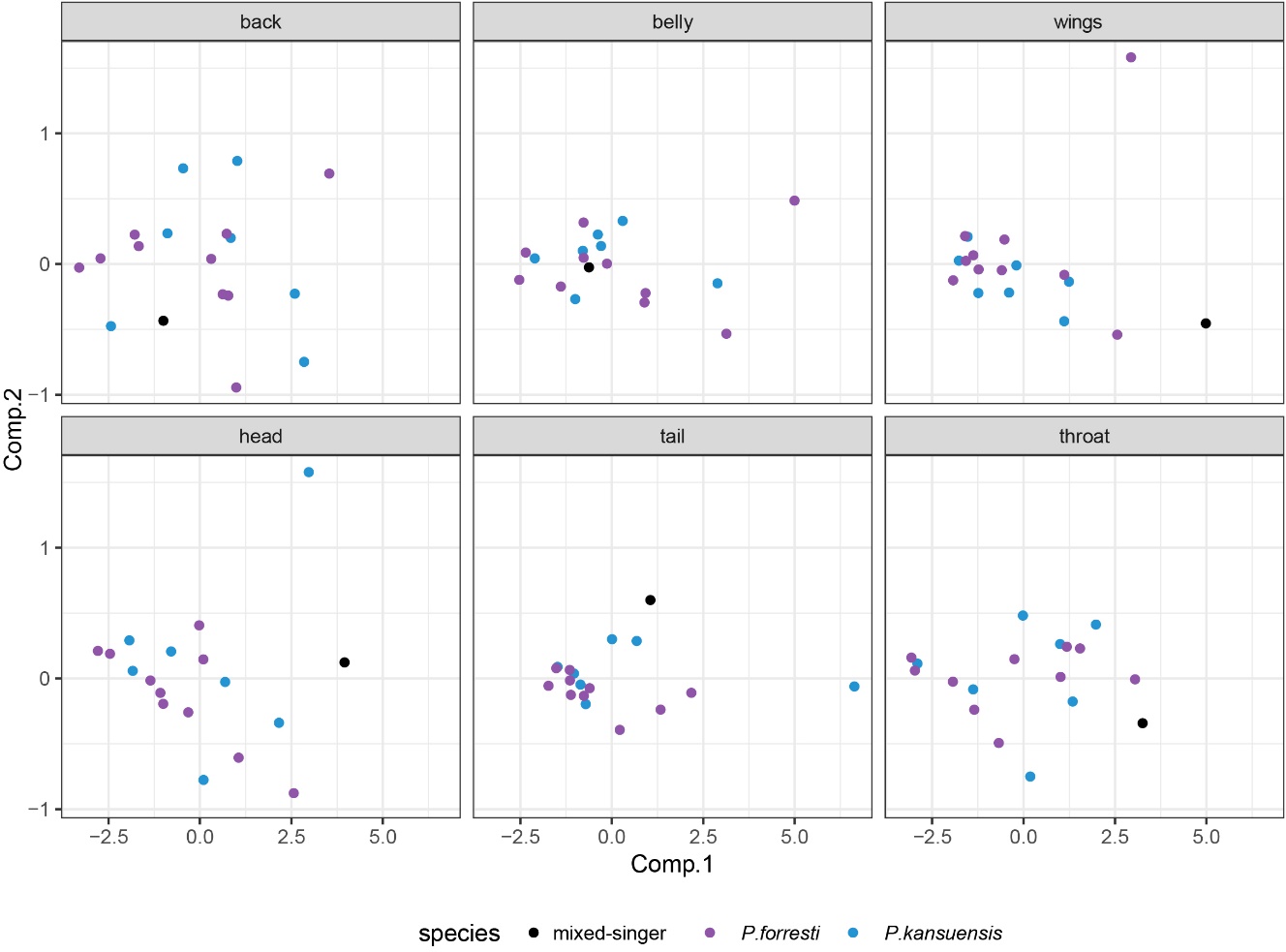
**

**Supplementary figure 3.** Complete verses and units of *Phylloscopus forresti* in allopatric areas and the contact zone. a-e: 6 complete verses of *P. forresti* in allopatric areas. a and b: Jiangqun Forest Farm, Qinghai; c: Hebei, Qinghai; d: Zhagana, Gansu; e: Foping, Shaanxi. g-m: 7 complete units of *P. forresti* in allopatric areas. g-i: Foping, Shaanxi; j-k: Malu, Gansu; l: Dege, Sichuan; m: Nangqian, Qinghai. f, n and o: a complete verse and 2 complete units of *P. forresti* in the contact zone in Qiagai, Gansu.


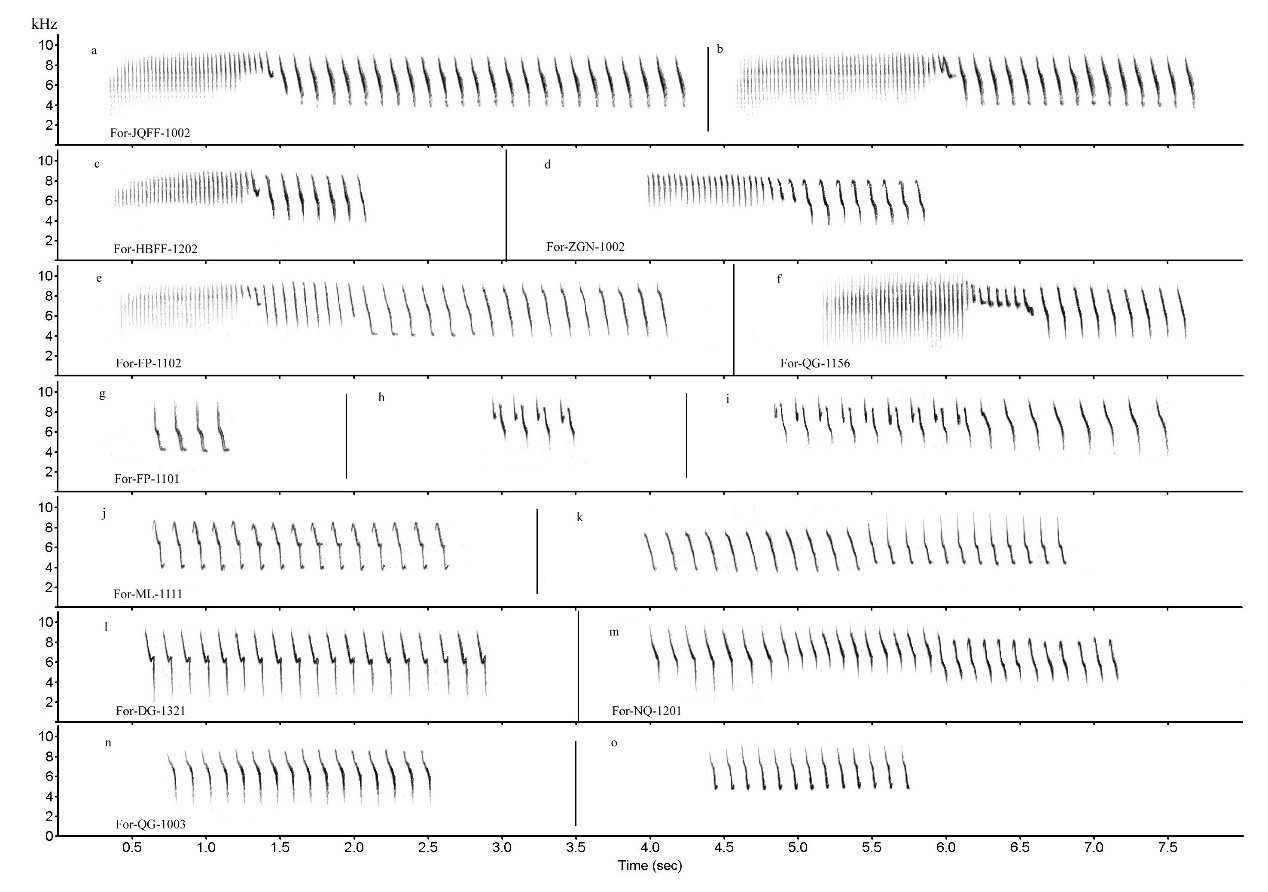


**Supplementary figure 4.** PCA of *P. forresti* song types based on log-transformed data.


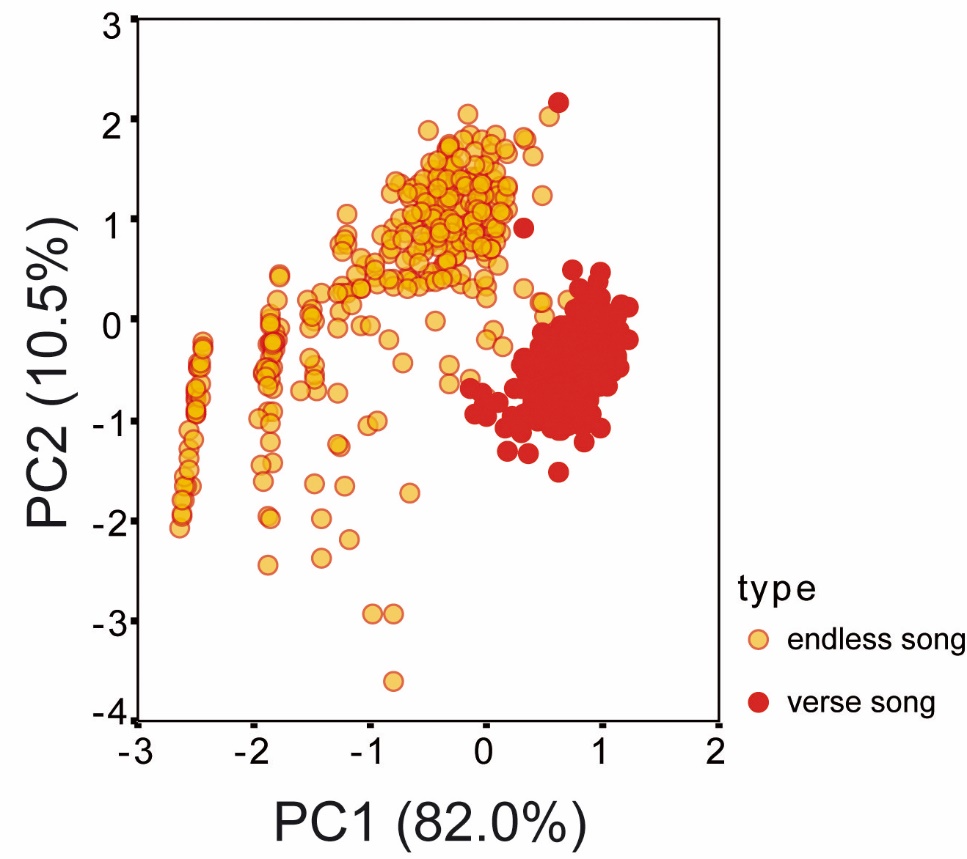


**Supplementary figure 5.** Complete verses of *Phylloscopus kansuensis* in allopatric areas and contact zone. a-e: 5 complete verses of *P. kansuensis* in allopatric areas. a and b: Qilian, Qinghai; c: Xinglongshan，Gansu; d: Lianhuashan, Gansu; e: Taizishan, Gansu; f-h: 3 complete verses of *P. kansuensis* in contact zone. f and g: Hezuo, Gansu; h: Qiagai，Gansu.

**
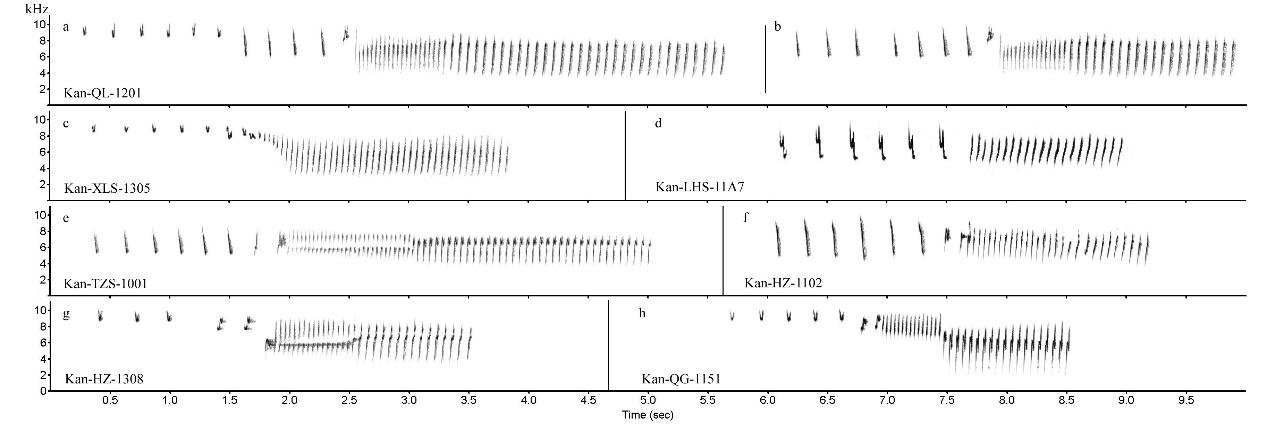
**

**Supplementary figure 6.** Complete verse songs of mixed-singers. a-b Hezuo, Gansu; c-d Qiagai, Gansu. a and c: normal verse song of *P. kansuensis*; b and d: mixed-song.


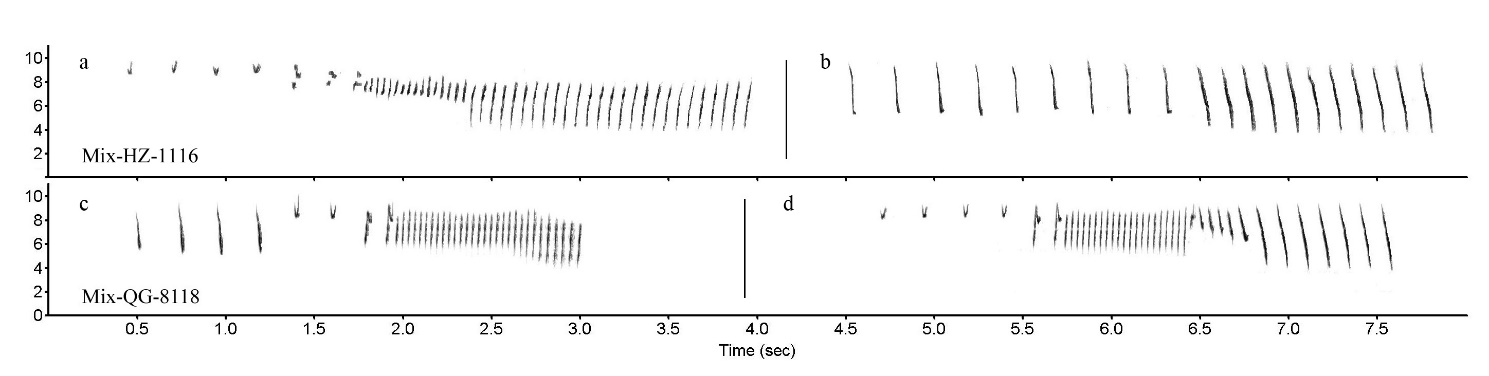


**Supplementary figure 7.** The accumulation curve of heterospecific songs of four mixed-singers. For simplicity, we treated typical *P. kansuensis* verse songs as conspecific songs. Mixed-songs and *P. forresti* songs were treated as heterospecific songs. We counted the proportion of heterospecific songs in the continuous recordings of mixed-singers. The value in x-axis indicates the first n recordings were considered.

**
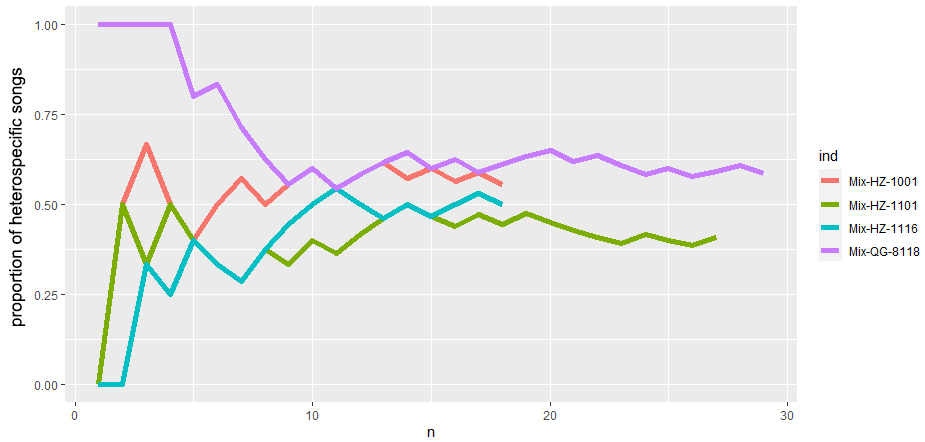
**

**Supplementary figure 8.** Distribution of three different song types of *P. kansuensis.*


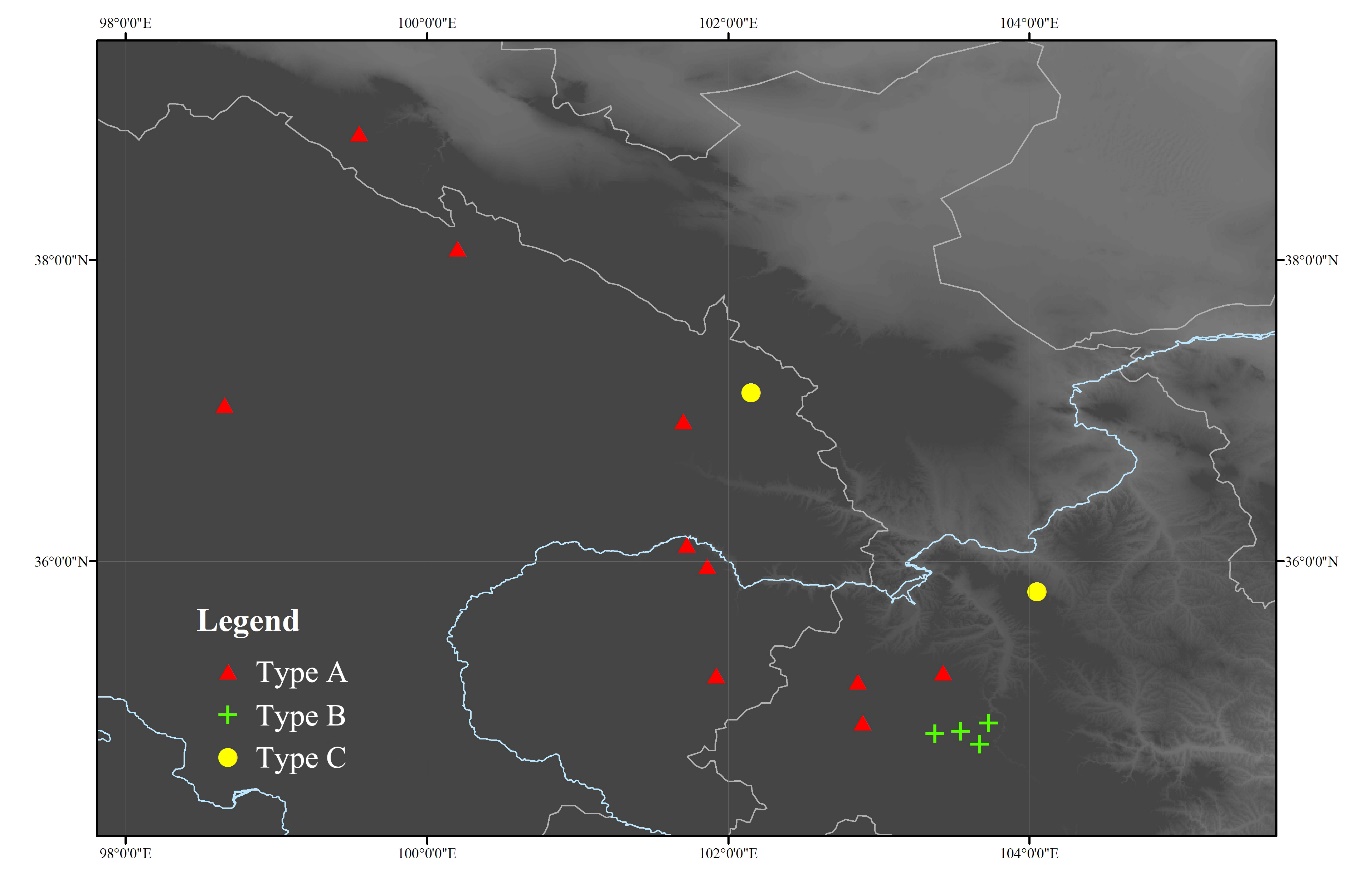


**Supplementary figure 9.** Song types (high-low) of *P. kansuensis*. A) high and low types of *P. kansuensis*. B) PCA based on original mean values. C) PCA based on log-corrected values.


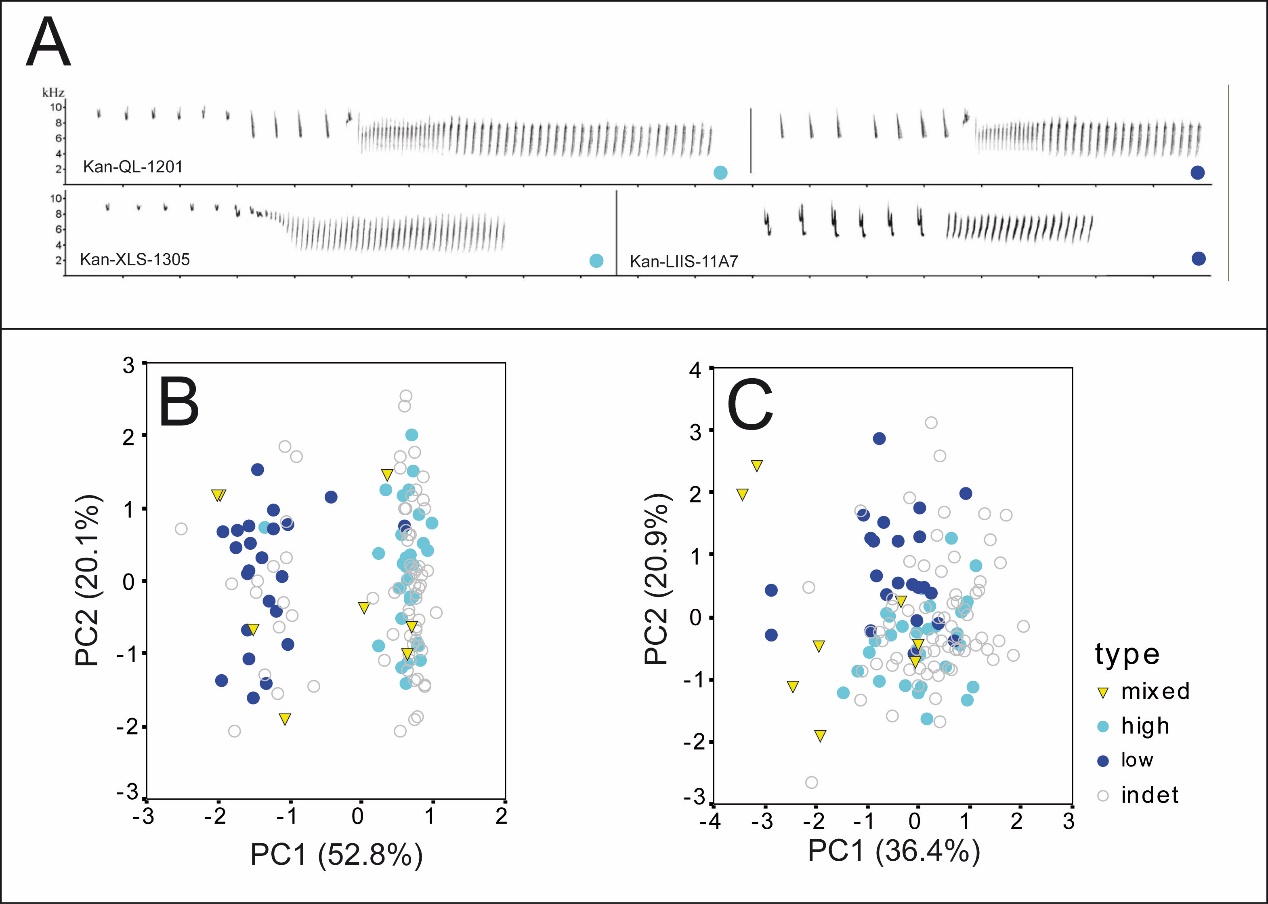


**Supplementary figure 10.** Median-joining haplotype network based on 758 bp cyt*b* (n= 99)*.*


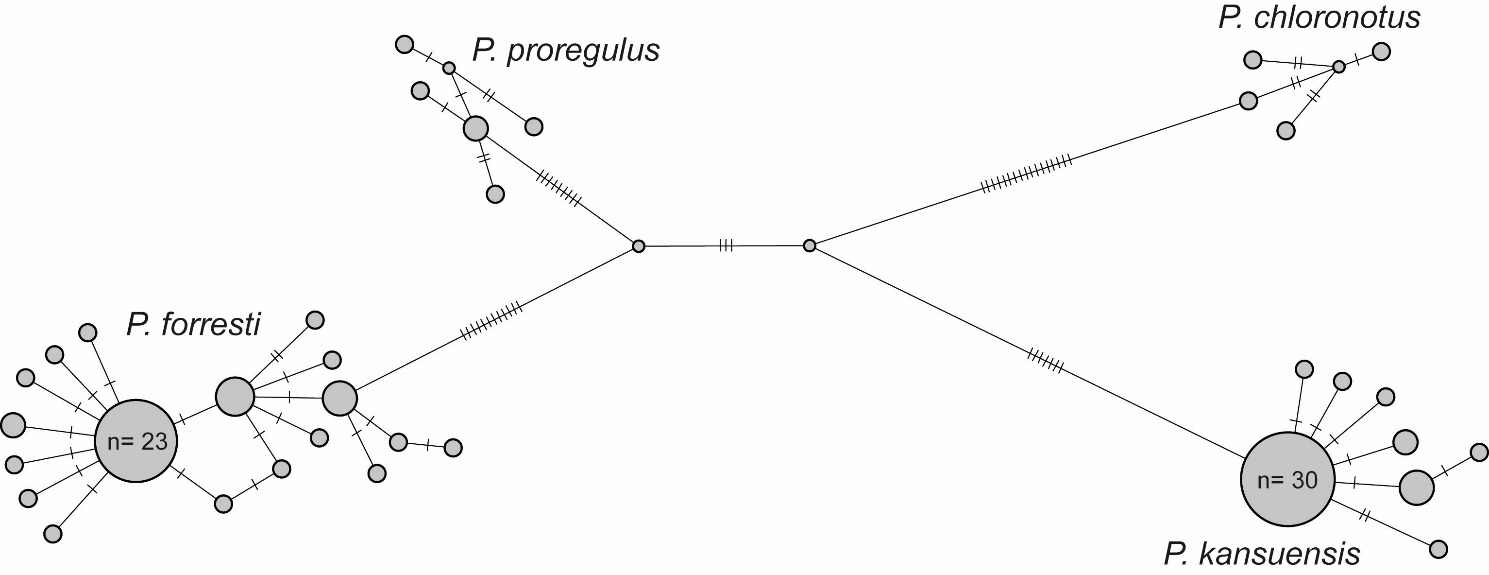


**Supplementary figure 11.** Fst of windows (50 KB) in sympatric and allopatric populations.


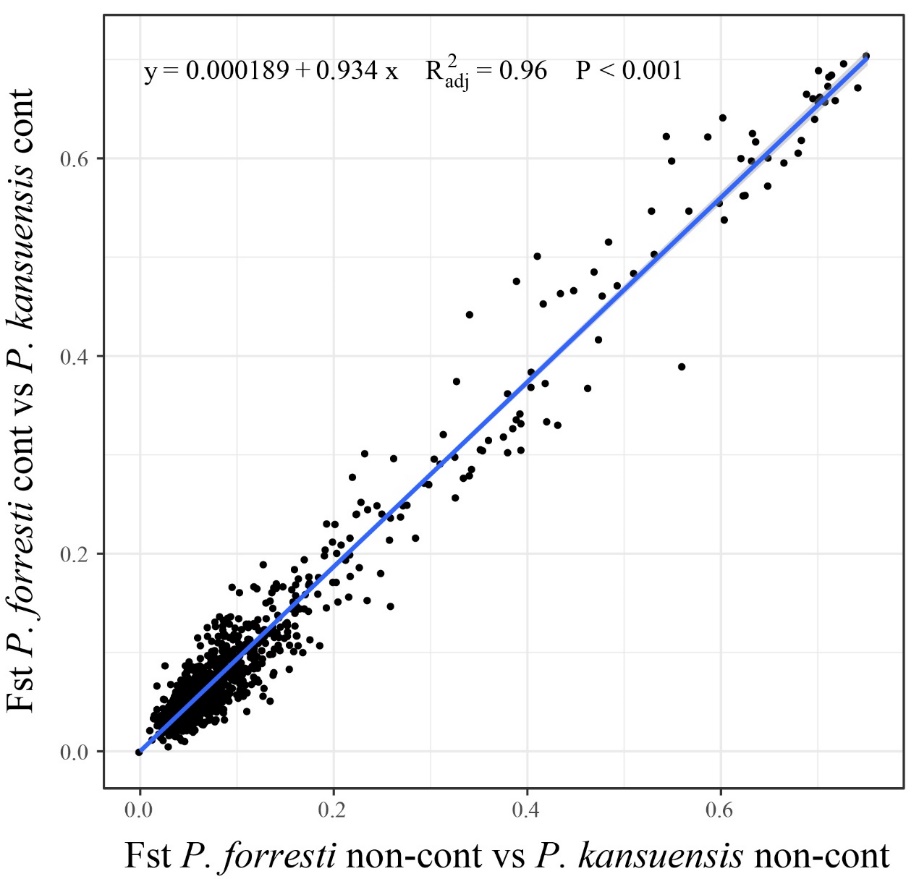


**Supplementary figure 12.** The linkage disequilibrium decline illustrated by R^2^.


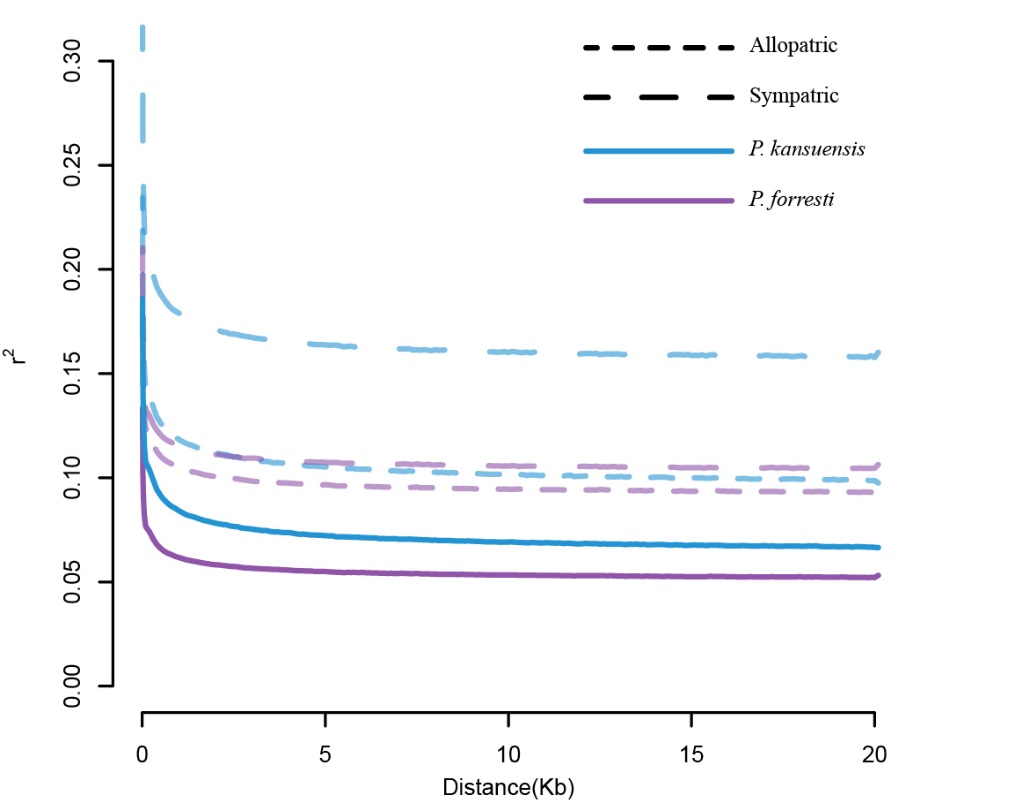


**Supplementary figure 13.** Population demography of *P. forresti* and *P. kansuensis*.


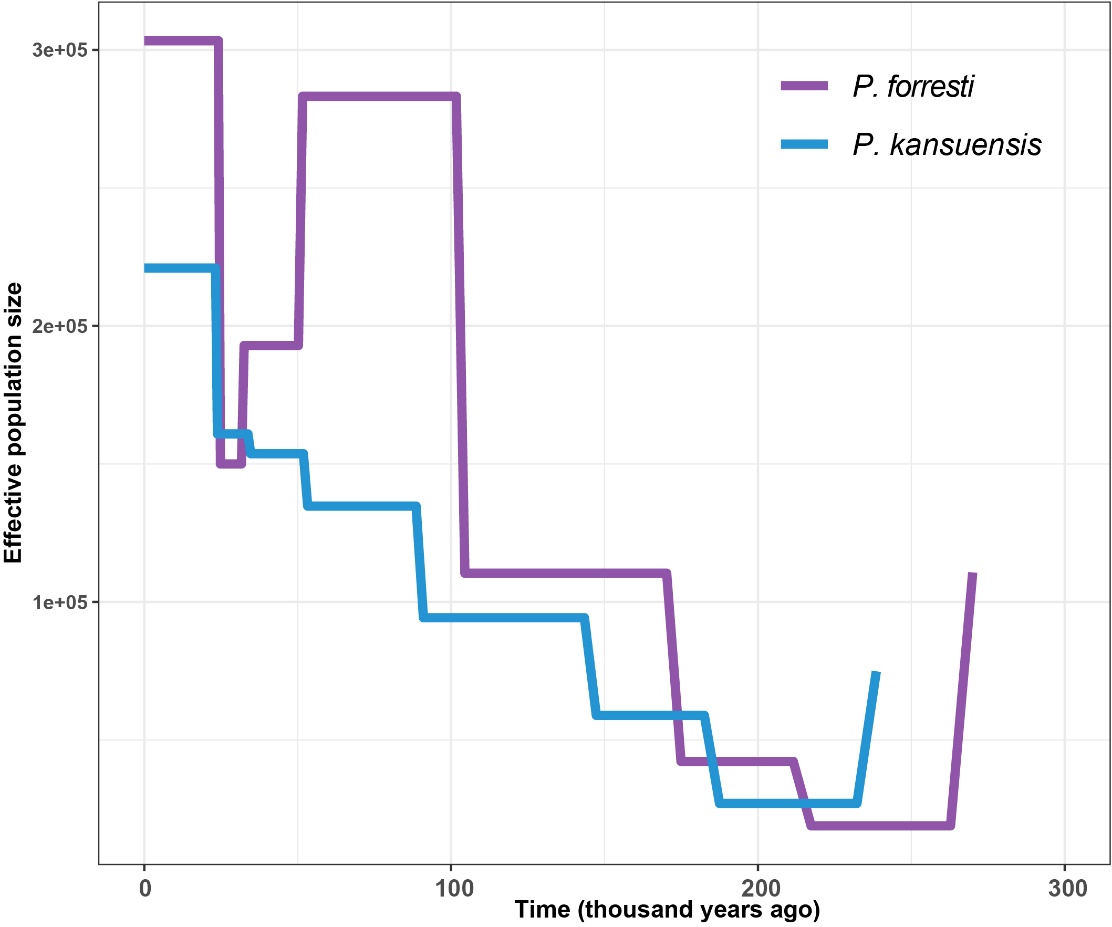


**Supplementary figure 14.** Venn diagram of the datasets. Cyt*b*48 is not included since it is a subset of genomic dataset.


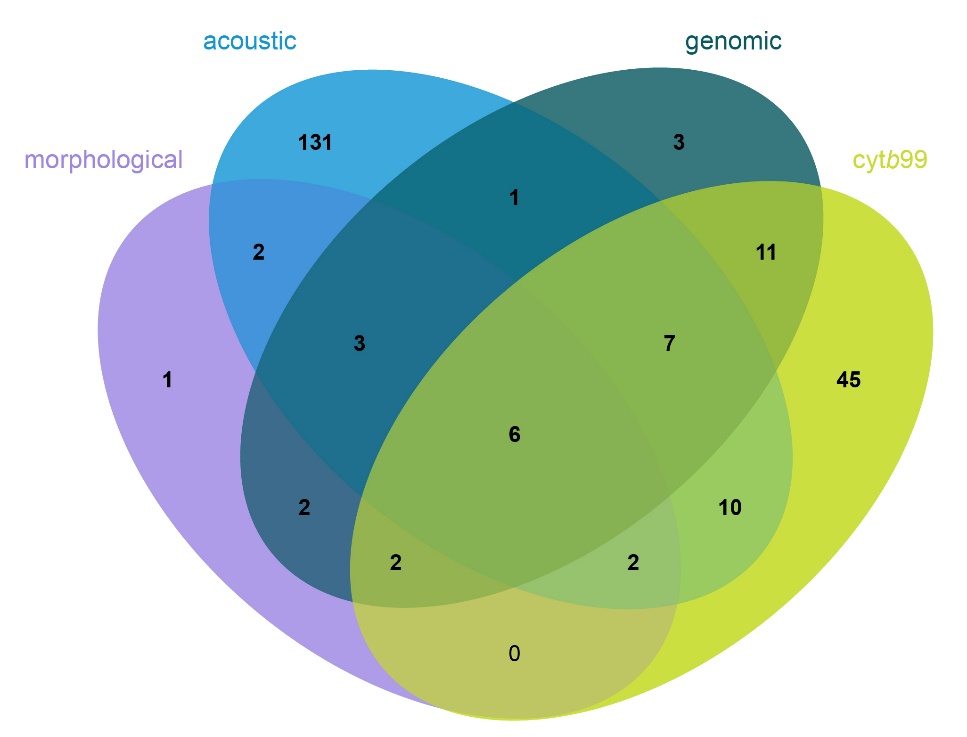

Supplement: msad053_Supplementary_Data [file msad053_supplementary_data.zip › MBE-22-0664.R3.supp_figs.docx]
